# Targeting pathological cells with senolytic drugs reduces seizures in neurodevelopmental mTOR-related epilepsy

**DOI:** 10.1038/s41593-024-01634-2

**Published:** 2024-05-06

**Authors:** Théo Ribierre, Alexandre Bacq, Florian Donneger, Marion Doladilhe, Marina Maletic, Delphine Roussel, Isabelle Le Roux, Francine Chassoux, Bertrand Devaux, Homa Adle-Biassette, Sarah Ferrand-Sorbets, Georg Dorfmüller, Mathilde Chipaux, Sara Baldassari, Jean-Christophe Poncer, Stéphanie Baulac

**Affiliations:** 1 Sorbonne Université, Institut du Cerveau - Paris Brain Institute - ICM, Inserm, CNRS, AP-HP, Hôpital de la Pitié Salpêtrière, Paris, France; 2 Institut du Fer à Moulin, INSERM, Sorbonne Université, UMR-S 1270, Paris, France; 3 Service de Neurochirurgie, AP-HP, Hôpital Lariboisière, Paris, France; 4 GHU Paris, Psychiatrie et Neurosciences, Paris, France; 5 Université de Paris Cité, Service d’Anatomie Pathologique, AP-HP, Hôpital Lariboisière, DMU DREAM, UMR 1141, INSERM, Paris, France; 6 Department of Pediatric Neurosurgery, Rothschild Foundation Hospital, Paris, France; 7 Present Address: Department of Basic Neurosciences, University of Geneva, Geneva, Switzerland; 8 Present Address: NeuroNA Human Cellular Neuroscience Platform, Fondation Campus Biotech Geneva, Geneva, Switzerland

**Keywords:** Epilepsy, Epilepsy, Senescence

## Abstract

Cortical malformations such as focal cortical dysplasia type II (FCDII) are associated with pediatric drug-resistant epilepsy that necessitates neurosurgery. FCDII results from somatic mosaicism due to post-zygotic mutations in genes of the PI3K-AKT-mTOR pathway, which produce a subset of dysmorphic cells clustered within healthy brain tissue. Here we show a correlation between epileptiform activity in acute cortical slices obtained from human surgical FCDII brain tissues and the density of dysmorphic neurons. We uncovered multiple signatures of cellular senescence in these pathological cells, including p53/p16 expression, SASP expression and senescence-associated β-galactosidase activity. We also show that administration of senolytic drugs (dasatinib/quercetin) decreases the load of senescent cells and reduces seizure frequency in an *Mtor*^S2215F^ FCDII preclinical mouse model, providing proof of concept that senotherapy may be a useful approach to control seizures. These findings pave the way for therapeutic strategies selectively targeting mutated senescent cells in FCDII brain tissue.

## Abstract (web summary)

Brain tissue from patients with focal cortical dysplasia type II (FCDII) exhibits dysmorphic neurons bearing hallmarks of senescence. Treatment with a senolytic drug reduces seizures in an FCDII preclinical mouse model.

## Main

Epilepsy is a common neurological disease characterized by recurrent, spontaneous seizures resulting from hypersynchronous electrical discharges of neuronal networks. Cortical malformations, including focal cortical dysplasia type II (FCDII), are common causes of pediatric epilepsy and developmental delay^1^. FCDII-associated epilepsy is typically resistant to anti-seizure medication, necessitating surgical resection of the epileptogenic zone, and is the most prevalent cortical malformation in pediatric epilepsy surgery^2^. FCDII is characterized by localized cortical dyslamination and the presence of pathological cytomegalic cells, namely neurofilament-accumulating dysmorphic neurons (DNs) (present in FCDIIa and FCDIIb) and, in some cases, balloon cells (BCs), which are enlarged glossy cells expressing both glial and neuronal markers (present in FCDIIb only)^3^. Identifying the molecular alterations and targetable biomarkers of DNs and BCs is key to understanding the pathogenesis of FCDII and developing targeted treatments.

Post-zygotic/somatic variants arise along the zygote-to-adult developmental trajectory, resulting in genetic mosaicism^4^. Pathogenic somatic mutations occurring during cortical development have emerged as important causes of FCDII (reviewed in refs. ^5,6^). FCDII lesion size primarily reflects the developmental stage at which the somatic mutation arises. Hence, the proportion of mutated cells varies from less than 1% of the resected cells in small FCDII to more than 20% in those involving an entire hemisphere, as in hemimegalencephaly (HME). Somatic mutations are typically found in the canonical PI3K-AKT-mTOR signaling cascade genes (*AKT3*, *DEPDC5*, *MTOR*, *PIK3CA*, *RHEB*, *TSC1* and *TSC2*) and lead to a cellular mosaic pattern of mTOR-hyperactive cells intermingled with normal-appearing neurons^7–9^. DNs and BCs carry the mTOR-activating mutations, establishing a causal link among the genetic mutation, hyperactivation of the mTOR pathway and the generation of cytomegalic cells^8,10^.

FCDII belongs to the spectrum of neurological diseases referred to as ‘mTORopathies’, which encompasses tuberous sclerosis complex (TSC), a multi-systemic genetic disorder resulting from germline mutations in *TSC1* or *TSC2* genes^11^. The mTOR signaling cascade is crucial for many biological processes, including cell growth, cell survival, autophagy and metabolism through lysosome and mitochondria biogenesis^12^. In the brain, mTOR hyperactivation leads to abnormal neuronal differentiation, migration and increased dendritic arborization (reviewed in refs. ^13,14^).

In the present study, we investigated the molecular, cellular and electrophysiological features of abnormal FCDII cells in acute human cortical slices and genetic mouse models. We report an association between the density of DNs carrying an mTOR-activating mutation and tissue epileptogenicity in human FCDII in vitro recordings. Furthermore, we discovered that abnormal cytomegalic cells display a senescence-like phenotype, both in FCDII surgical specimens and mouse models of mTOR hyperactivation. Finally, we show that administrating senolytic drugs, which selectively target senescent cells, in a preclinical mouse model of FCDII reduces the occurrence of epileptic seizures.

## Results

### Epileptiform activity correlates with DN density

**Fig. 1:**
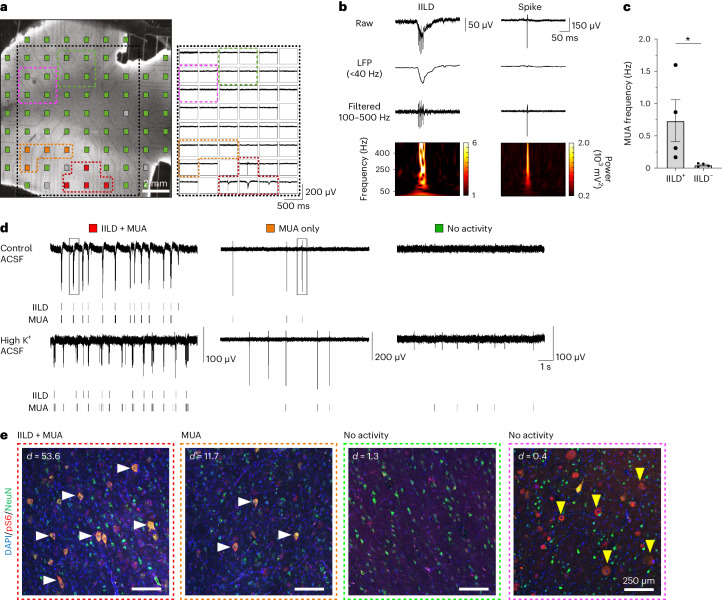
Spontaneous interictal-like activity and dysmorphic neuron topography in human FCDIIb brain cortical slices. **a**, Left, micrograph of a cortical slice of resected brain tissue from a patient with FCDIIb (patient ID 8, slice 3) showing MEA layout. Electrodes are color-coded to indicate the type of activity detected (red: IILDs and MUA; orange: MUA only; green/pink: no activity; gray: defective electrodes). Right, representative recording of the simultaneous activity on the electrodes located within the area delimited by the black dotted lines. Recordings from electrodes with IILDs+MUA or MUA only are outlined and color-coded as in the left panel. **b**, Top, representative traces of the raw signal of an IILD (left) or a spike (right) from the two boxed portions of recordings shown in **d**. The signal is represented after low-pass filtering (<40 Hz) to reveal the slow component of the IILD or band-pass filtering (100–500 Hz) to reveal spikes and high-frequency oscillations. Spectrograms of the raw signals (bottom) show that the IILD includes high-frequency components superimposed on a slow oscillation, whereas the spike shows only a sharp, high-frequency component. **c**, Mean MUA frequency recorded in slice 3 from *n* = 4 electrodes displaying IILDs (IILD^+^) and *n* = 4 without IILDs (IILD^−^). *P = 0.0286, two-tailed Mann–Whitney rank-sum test. **d**, Representative recordings from color-coded electrodes shown in **a**, with corresponding raster plots of IILDs and MUA. Recordings are shown in control ACSF and after switching to high-K^+^ ACSF to promote neuronal activity. Note that MUA increases in all three recorded areas, whereas IILDs remain restricted to the electrode that previously displayed them. **e**, Immunofluorescent stainings from slice 3 with antibodies against pS6 (red) and NeuN (green) in areas delineated by color-coded dashed lines in **a**. White horizontal arrows indicate DNs (NeuN^+^/pS6^+^) and yellow vertical arrows indicate BCs (NeuN^−^/pS6^+^). density (*d*) = number of dysmorphic neurons per mm^2^. Scatter dot plots are presented as mean ± s.e.m. LFP, local field potential. Source data

**Extended Data Fig. 1:**
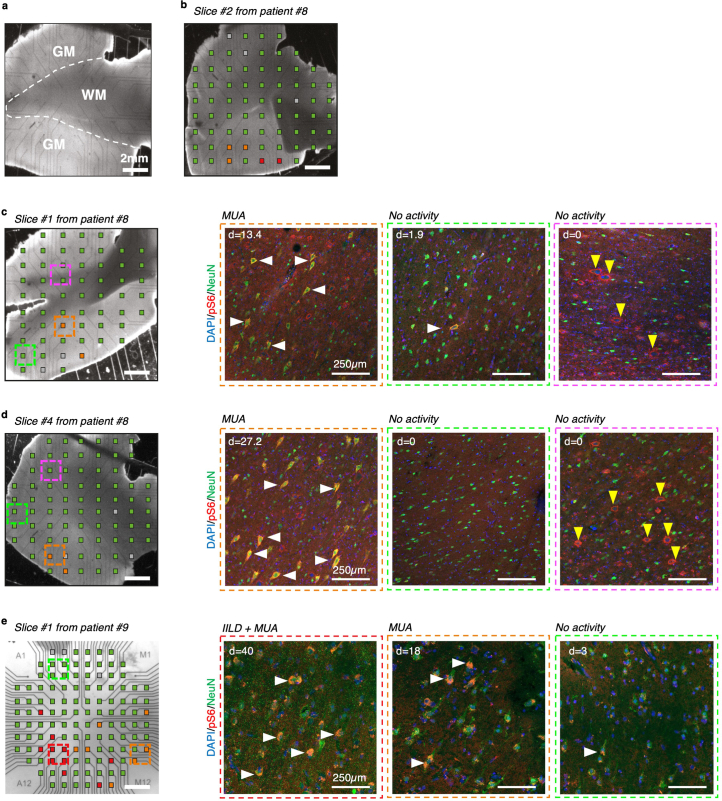
Cortical activity and dysmorphic neuron topography in human FCDIIb brain cortical slices. (**a**) Representative delineation of gray-white matter boundary. GM: gray matter, WM: white matter. (**b-e**) (Left) Micrographs of cortical slices of resected tissue from FCDIIb patients showing multielectrode array (MEA) layout. Electrodes are color-coded to show the type of activity detected (Red: IILD + MUA; Orange: MUA only; Green: no activity; Gray: damaged electrode). Scale bar: 2 mm. (Right) Immunofluorescent staining from the corresponding cortical slice with antibodies against pS6 (red) and NeuN (green) in regions delineated by color-coded dashed lines on MEA layout. Dysmorphic neurons (DNs) are identified by horizontal white arrows and balloon cells (BCs) are identified by vertical yellow arrows. d: density = number of DNs/mm^2^. Cortical slices represented are: (b) Slice #2 from patient ID#8 (for which no immunofluorescent staining is available); (c) Slice #1 from patient ID#8; (d) Slice #4 from patient ID#8; (e) Slice #1 from patient ID#9. MEA recordings and histology stainings on the same sections could only be performed once on each slice. Source data

We performed extracellular recordings using multi-electrode arrays (MEAs) in four adjacent acute cortical slices from a surgical FCDII cortical gyrus (patient ID 8 with p.Ala1459Pro hotspot somatic *MTOR* mutation and neuropathology diagnosis confirming the presence of DNs and BCs) (Fig. 1a–d and Extended Data Fig. 1a–d).

Cortical slices display two main types of activities (Fig. 1a,b): (1) multi-unit activity (MUA) with individual spikes and spike bursts and (2) interictal-like discharges (IILDs), consisting of slower, often biphasic local field potential deflections with low-frequency (<40 Hz) and high-frequency (100–500 Hz) oscillatory components (Fig. 1b). Synchronous IILDs were recorded on neighboring electrodes (Fig. 1a), suggesting that they reflect network-generated events. On all slices, MUA was detected from 2–7 neighboring electrodes, delineating an area of gray matter with localized activity (Fig. 1a and Extended Data Fig. 1a–d). Spontaneous IILDs were detected on 2–4 electrodes in slices 2 and 3 (Fig. 1a and Extended Data Fig. 1b). MUA was significantly higher on electrodes where IILDs were detected compared to neighboring electrodes without IILDs (slice 3, 0.74 ± 0.32 versus 0.05 ± 0.01 Hz) (Fig. 1c), suggesting that the IILD-generating zone was associated with higher neuronal excitability. No spontaneous activity was detected on most electrodes (54–71), encompassing areas of both gray and white matter (Fig. 1a,d and Extended Data Fig. 1b–d). MEA recordings, therefore, demarcate a gray matter area characterized by an activity gradient, with a restricted zone displaying both IILDs and MUA and a surrounding area with only spontaneous MUA or no activity. Furthermore, switching to a pro-convulsive extracellular solution (6 mM K^+^, 0.25 mM Mg^2+^) increased MUA across all recorded areas (Fig. 1d, bottom), whereas IILDs remained restricted to the same area as in control conditions, suggesting that IILD generation reflects specific local network properties. In a second patient (ID 9 with p.Glu17Lys *AKT3* hotspot somatic variant and FCDIIb neuropathological diagnosis with DNs and BCs), we also detected spontaneous abnormal activity (IILDs and MUA) in a defined cortical area of the slice (Extended Data Fig. 1e).

We next asked whether the gradient of cortical hyperactivity correlated with the presence of DNs and BCs near the recording electrodes. For this purpose, we performed immunofluorescence staining against phosphorylated-S6 (pS6) protein (a standard readout for mTOR activity) and NeuN (neuronal marker) to ascertain DNs as cytomegalic pS6^+^/NeuN^+^ neurons on four of the recorded slices. By overlaying whole-mount immunofluorescence images and bright-field images of MEA recordings, we observed a correlation between neuronal activity and the presence of cytomegalic pS6^+^ DNs. This correlation was observed in all slices from both patients (ID 8 and ID 9), with densities varying from 53.6 DNs per mm^2^ in regions displaying spontaneous IILDs to 11.7 DNs per mm^2^ in areas exhibiting MUA only (Fig. 1e and Extended Data Fig. 1c–e). Dysmorphic pS6^+^ neurons were not observed in the electrically silent areas of the brain slices. No spontaneous IILDs or MUAs were recorded in the area containing BCs, identified as cytomegalic round-shaped pS6^+^/NeuN^−^ cells, which were located at the gray–white matter boundary distant from the area containing DNs (Fig. 1a,d,e and Extended Data Fig. 1c–e). Together, these data suggest that cytomegalic DNs (neurons carrying the mutation) may contribute to epileptiform activity, providing a rationale to selectively silence these cells to prevent seizures.

### FCDII pathological cells display a senescence phenotype

We then searched for targetable biomarkers of FCDII to identify novel therapeutic strategies. The mTOR pathway is implicated in cellular senescence and senescence-associated secretory phenotype (SASP) regulation^15,16^. Moreover, a study reported that BCs exhibit cell cycle arrest akin to premature cellular senescence^17^. Cellular senescence is a cell state characterized by a cell cycle arrest via tumor suppressor p53 and/or cyclin-dependent kinase p16 pathways, acquisition of SASP, loss of nuclear envelope integrity and increased lysosomal content detected by senescence-associated β-galactosidase (SAβGal) activity^18^. Senescent cells upregulate pro-survival pathways, resisting apoptosis; however, they are heterogeneous across cell types, tissues or diseases^19^.

**Fig. 2:**
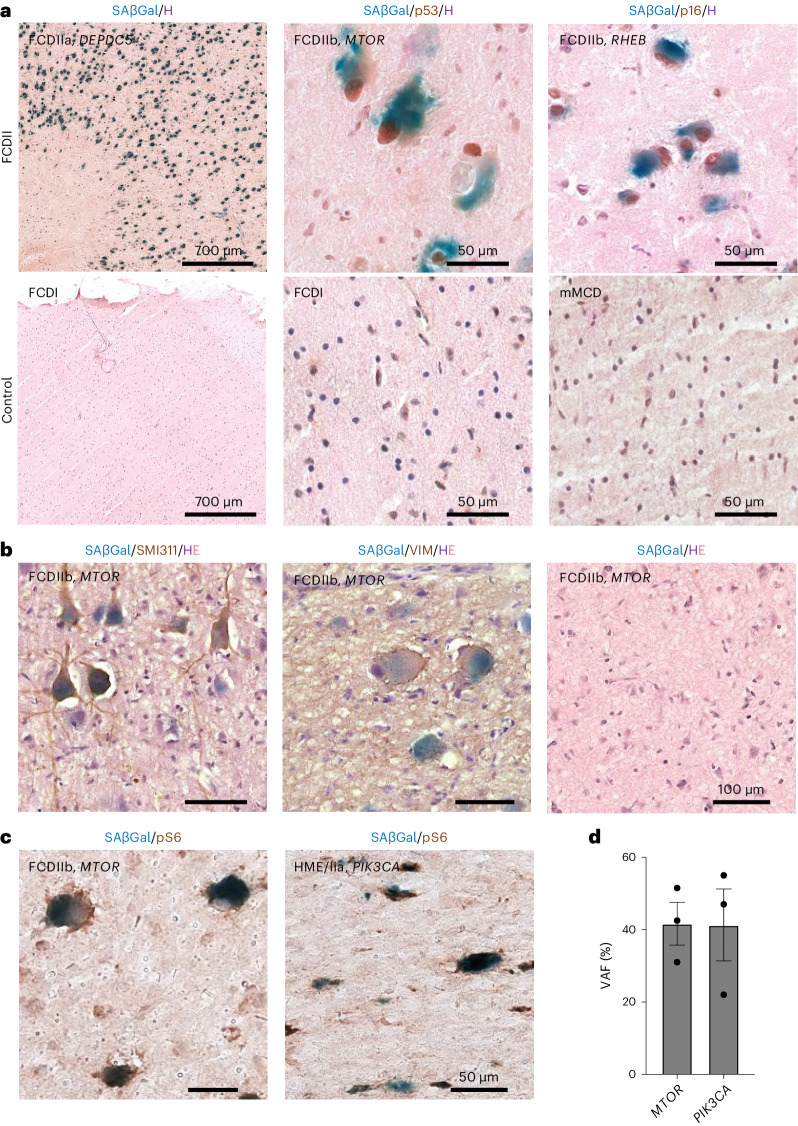
Cellular senescence hallmarks in FCDII surgical tissues. **a**, Representative SAβGal colorimetric assay (blue), p53 (brown) or p16 (brown) immunohistochemistry and hematoxylin (H; purple) counterstaining on FCDII samples (left to right: patient ID 10 (wider field of view), patient ID 3 and patient ID 14 and epilepsy control samples (left to right: patient ID 15 (wider field of view), patient ID 15 and patient ID 16)). **b**, SAβGal colorimetric assay (blue), SMI311 (brown) or VIM (brown) immunohistochemistry and hematoxylin and eosin (H, purple; E, pink) counterstaining on a FCDIIb sample (patient ID 8, slice 3) used for MEA recordings in a region with SMI311^+^ DNs (left), VIM^+^ BCs (middle) or without pathogenic cells (right). **c**, SAβGal colorimetric assay (blue) and pS6 (brown) immunohistochemistry on (left) *MTOR*-related FCDIIb (patient ID 3) and (right) *PIK3CA*-related HME/IIa (patient ID 13). **d**, VAF in *n* = 3 pools of *n* = 70–80 microdissected SAβGal^+^/pS6^+^ cytomegalic cells per pool from one *MTOR*-related FCDIIb (patient 3) and one *PIK3CA*-related HME/IIa (patient ID 13). Each dot indicates a biological replicate. Scatter dot plots are presented as mean ± s.e.m. Source data

**Extended Data Fig. 2:**
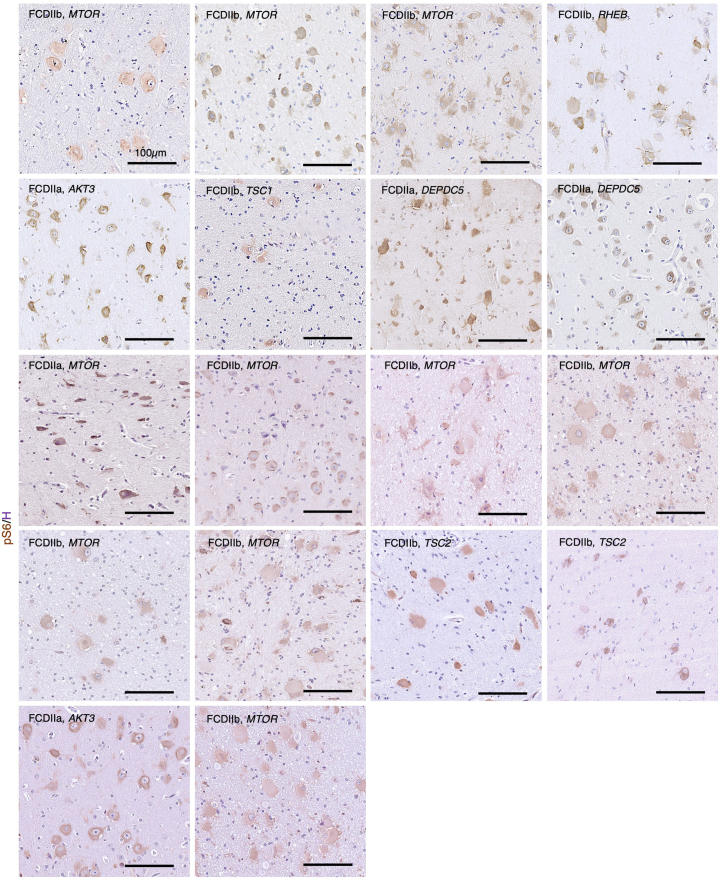
mTOR pathway activity in FCDII epileptic surgical tissues. Representative pS6 (brown) immunohistochemistry and hematoxylin (H; purple) counterstaining on n = 18 FCDII/HME samples (left to right, top to bottom: ID#6, 3, 5, 14, 12, 1, 2, 10, 21, 22, 23, 24, 25, 26, 27, 28, 29, 30). Scale bar: 100um.

**Extended Data Fig. 3:**
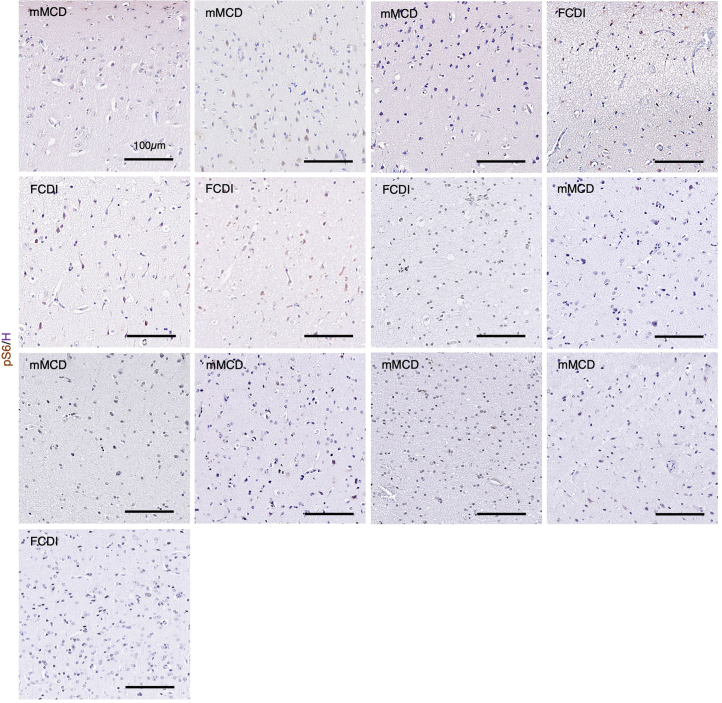
mTOR pathway activity in control epileptic surgical tissues. Representative pS6 (brown) immunohistochemistry and hematoxylin (H; purple) counterstaining on n = 13 control epileptic resected brain tissues negative for mTOR-pathway genes mutations by gene-panel deep-sequencing (left to right, top to bottom: ID#16, 18, 20, 15, 17, 19, 31, 32, 33, 34, 35, 36, 37). Scale bar: 100um.

**Extended Data Fig. 4:**
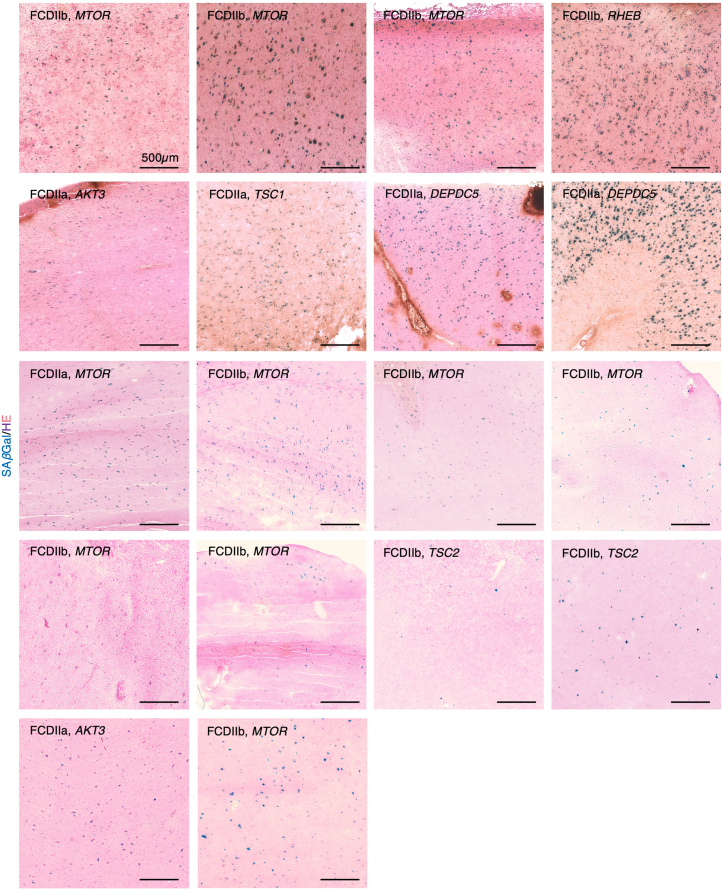
SA*β*Gal colorimetric assay in FCDII epileptic surgical tissues. Representative images of SAβGal colorimetric assay and HE coloration on n = 18 FCDII/HME samples carrying pathogenic variants in MTOR-pathway genes (left to right, top to bottom: ID#6, 3, 5, 14, 12, 1, 2, 10, 21, 22, 23, 24, 25, 26, 27, 28, 29, 30). Scale bar: 500um.

**Extended Data Fig. 5:**
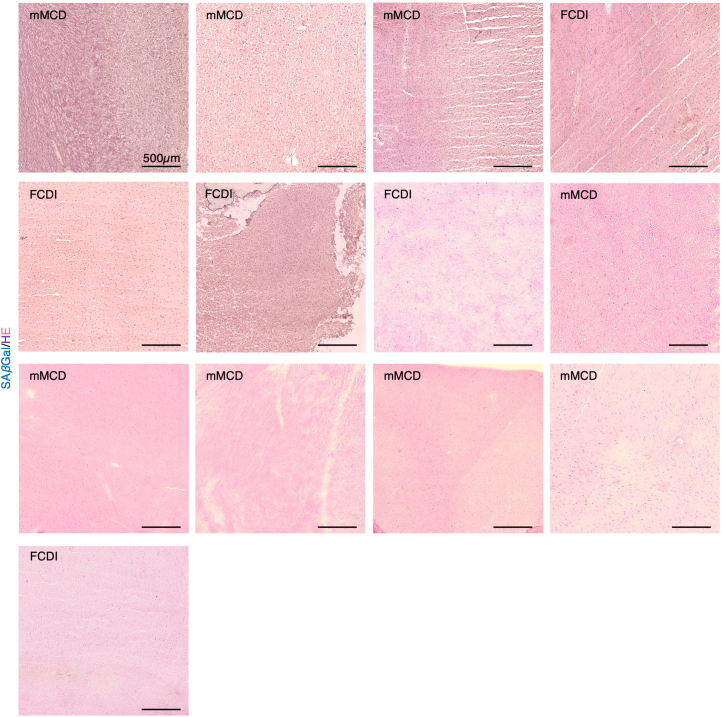
SA*β*Gal colorimetric assay in control epileptic surgical tissues. Representative images of SAβGal colorimetric assay and HE coloration on n = 13 control epileptic resected brain tissues negative for mTOR-pathway genes mutations by gene-panel deep-sequencing. (left to right, top to bottom: ID#16, 18, 20, 15, 17, 19, 31, 32, 33, 34, 35, 36, 37).

We thus investigated cellular senescence histologically in 22 surgical brain tissues obtained from pediatric FCDII patients with somatic mutations in the PI3K-AKT-mTOR pathway genes (*AKT3*, *DEPDC5*, *MTOR*, *PIK3CA*, *RHEB*, *TSC1* and *TSC2*) and 13 epilepsy control patients (non-mTOR-related cortical malformation). Patients were selected to ensure a homogenous cohort in terms of age at seizure onset, age at surgery, duration of epilepsy, seizure frequency and surgical outcome. Clinical, neuropathological and genetic characteristics of all cases are detailed in Table 1 and Supplementary Tables 1 and 2. In all FCDII samples, DNs and BCs displayed mTOR hyperactivity as revealed by pS6 immunostaining, which was never observed in epileptic control tissues (Extended Data Figs. 2 and 3). We performed a lysosomal SAβGal colorimetric assay along with immunostaining for p53 and p16 on frozen brain specimens, which are three canonical markers of cellular senescence (reviewed in ref. ^18^). In all FCDII tissues (both FCDIIa and FCDIIb subtypes) regardless of the mutated gene, strong SAβGal reactivity and p53^+^ and p16^+^ nuclei were consistently observed in most of the cytomegalic cells (as determined by their large soma diameter, >25 µm; SAβGal^+^p53^+^: 92 ± 1.7%; SAβGal^+^p16^+^: 85 ± 2.7%; *n* = 5 FCDII) (Fig. 2a and Extended Data Fig. 4). In contrast, senescence-associated markers were not detected in neighboring normal-sized cells of the same tissues nor in non-mTOR-related epileptic tissues (Fig. 2a and Extended Data Fig. 5), therefore delineating specific biomarkers of FCDII pathological cells.

**Table 1:** Clinical, neuropathological and genetic features of the patient cohort

Patient ID	Neuropathology	Gene	Mutation; VAF	Age at seizure onset	Age at surgery	Surgical topography
**Cases**						
1 (FCD-64)	FCDIIb	*TSC1*	p.E636fs*51; 3.7%	18 m	4 y	Frontal
2 (FCD-33)	FCDIIa	*DEPDC5*	2-hit: p.R286* (germline); Q289* (10% somatic)	1.2 y	6.75 y	Frontal
3 (FCD-56)	FCDIIb	*MTOR*	p.T1977K; 5.5%	8 m	16.1 y	Fronto-parietal
4 (FCD-57)	FCDIIb	*MTOR*	p.S2215F; 1.3%	4.5 m	9.1 y	Temporo-parietal
5 (FCD-59)	FCDIIb	*MTOR*	p.S2215F, 3.4%	16 m	13 y	Frontal
6 (FCD-61)	FCDIIb	*MTOR*	p.S2215Y; 3.7%	2 m	6.7 y	Parietal
7 (FCD-70)	FCDIIb	Panel-neg	N/A	2.5 y	16.1 y	Frontal
8	FCDIIb	*MTOR*	p.A1459P; 2%	18 m	16.8 y	Frontal
9	FCDIIb	*AKT3*	p.E17K; 1%	14 y	42 y	Frontal
10 (FCD-36)	HME/IIa	*DEPDC5*	2-hit: c.3021+1 G > A (germline); LOH (somatic)	1d	3 m	Frontal^a^
11 (HME-73)	HME/IIb	*MTOR*	p.A1459D; 9.2%	1d	3 m	Frontal^a^
12 (HME-74)	HME/IIa	*AKT3*	p.E17K; 12%	5d	1.3 y	Frontal^a^
13 (HME-77)	HME/IIa	*PIK3CA*	p.H1047R; 21.2%	15d	6 m	Frontal^a^
14 (HME-79)	HME/IIb	*RHEB*	p.Y35L; 17.6%	3d	5 m	Frontal^a^
21	FCDIIa	*MTOR*	p.S2215Y; 0.9%	2 m	7 y	Temporal
22	FCDIIb	*MTOR*	p.S2215F; 2%	6 m	2.1 y	Fronto-temporal
23	FCDIIb	*MTOR*	p.L1460P; 1.4%	5 y	12 y	Frontal
24	FCDIIb	*MTOR*	p.S2215Y; 4.8%	6.3 y	10 y	Frontal
25	FCDIIb	*MTOR*	p.S2215Y; 1.39%	20 m	8.5 y	Temporal
26	FCDIIb	*MTOR*	p.A1459D; 3.45%	13 m	5 y	Fronto-insular
27 (FCD-65)	FCDIIb	*TSC2*	p.R1743Q; 1.5%	2 y	4.75 y	Frontal
28	FCDIIb	*TSC2*	p.Q404*; 2%	2 m	11 m	Operculo-insular
29	FCDIIa	*AKT3*	p.E17K; 7.39%	15d	1.2 y	Frontal
30	FCDIIb	*MTOR*	p.A1459D; 1.93%	7 y	18 y	Frontal
**Epileptic controls**						
15 (FCD-16)	FCDI	Panel-neg	N/A	1.8 y	3.8 y	Temporal
16 (FCD-13)	mMCD	Panel-neg	N/A	1.7 y	10.5 y	Temporal
17 (FCD-18)	FCDI	Panel-neg	N/A	2 y	5.4 y	Occipital
18 (FCD-7)	mMCD	Panel-neg	N/A	5 m	5.7 y	Temporal
19 (FCD-12)	FCDI	Panel-neg	N/A	13 y	16.5 y	Frontal
20 (FCD-6)	mMCD	Panel-neg	N/A	2 m	1.9 y	Temporo-parietal
31 (FCD-17)	FCDI	Panel-neg	N/A	4 m	6 m	Temporal
32 (FCD-5)	mMCD	Panel-neg	N/A	11 y	14 y	Frontal
33 (FCD-8)	mMCD	Panel-neg	N/A	2 y	11.7 y	Insula
34 (FCD-15)	mMCD	Panel-neg	N/A	5 m	6.7 y	Parietal
35	mMCD	Panel-neg	N/A	8 m	3.9 y	Fronto-insular
36	mMCD	Panel-neg	N/A	4 y	10 y	Frontal
37 (FCD-10)	FCDI	Panel-neg	N/A	4 y	9.1 y	Occipital

IDs in parentheses refer to patients previously reported in Baldassari et al.^8^. LOH, loss of heterozygosity; m, months; mMCD, mild malformation of cortical development with excessive heterotopic neurons; N/A, not applicable; Panel-neg, panel-negative; y, years.

^a^Indicates cases in which a hemispherotomy (functional disconnection of one hemisphere) was performed. In all other cases, a resection was achieved.

We then asked whether all FCDII pathological cells presented cellular senescence features. First, we performed an SAβGal colorimetric assay together with immunohistochemistry against a canonical DN marker (non-phosphorylated neurofilament, SMI311) or a BC marker (vimentin (VIM)) in the same FCDII sample used for MEA recordings (patient ID 8, slice 2). Most of the DNs in the area with epileptiform activity displayed a strong reactivity to SAβGal (SAβGal^+^SMI311^+^: 88 ± 6.6%; *n* = 5 FCDII) as well as most of the BCs located distantly in the gray–white matter boundary (SAβGal^+^VIM^+^: 95 ± 2.7%; *n* = 5 FCDII; Fig. 2b). Next, we investigated whether SAβGal^+^ cells displayed increased mTOR activity and carried the pathogenic mTOR pathway-activating mutation. SAβGal colorimetric assay and pS6 immunohistochemistry confirmed that most of the SAβGal^+^ cells were pS6^+^ (89 ± 6.1%; *n* = 5 FCDII; Fig. 2c). We performed laser capture microdissection of SAβGal^+^pS6^+^ cells followed by droplet-digital PCR to accurately assess the variant allele frequency (VAF) on two FCDII tissues with somatic variants in *MTOR* and *PIK3CA*. The VAF was 42% and 41% in microdissected cells for *MTOR* and *PIK3CA* cases, respectively, in contrast to 5.5% and 21.2% in the bulk tissue. This result indicates a substantial enrichment of mutation load and suggests that the most SAβGal^+^ cells carry the mTOR-activating variant in a heterozygous state (Fig. 2d). It is consistent with our previous findings that somatic mutations are enriched in FCDII cytomegalic cells^8,10^.

**Extended Data Fig. 6:**
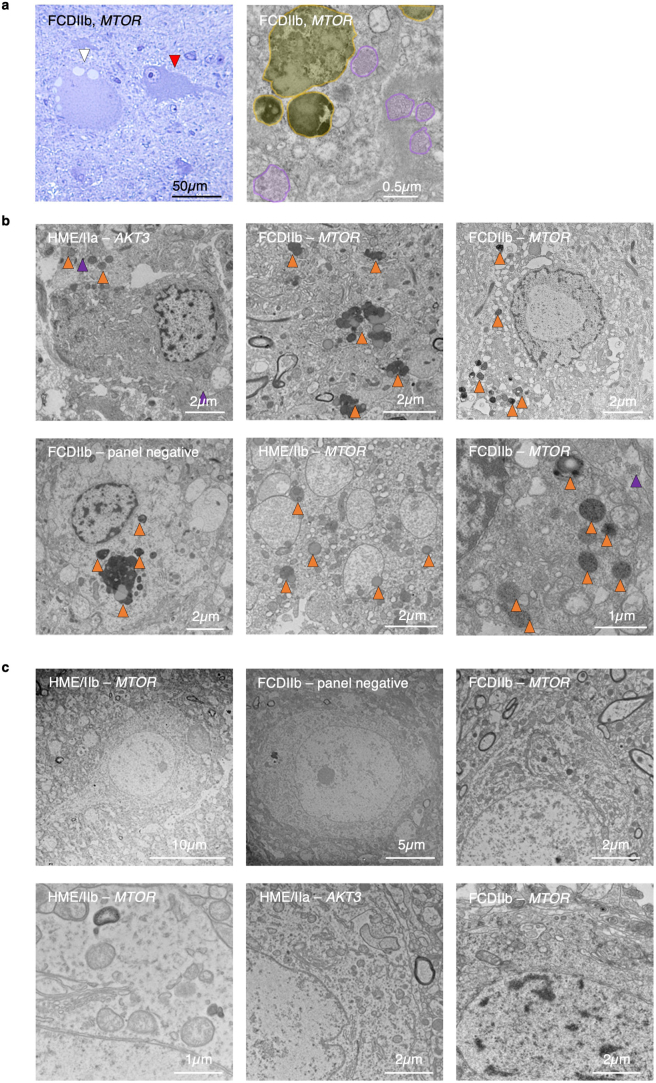
Macromolecular content in cytomegalic and normal-appearing FCDII cells. (**a**) (Left) Toluidine blue staining showing a cytomegalic cell with a soma diameter >25μm (white arrow) and a normal-appearing cell (red arrow) in an *MTOR*-related FCDIIb tissue (ID#3). (Right) Electron microscopy images of the intracellular content of a cytomegalic cell from the same tissue. Orange shade: lysosomes. Purple shade: multivesicular bodies. (**b**) Representative images of cytomegalic cells in n = 5 FCDII resected brain tissues showing abnormal accumulation of electron-dense enlarged lysosomes (orange arrows) and multivesicular bodies (purple arrows). Patient IDs (left to right, top to bottom): #12, 3, 4, 7, 11, 3. (**c**) Representative images of normal-appearing cells in the same panel of n = 6 FCDII cases showing typical cytoplasmic content. Patient IDs (left to right, top to bottom): #11, 7, 3, 11, 12, 3. Electron microscopy imaging was repeated at least three times.

Finally, we conducted ultrastructural examination by electron microscopy of five post-surgical FCDII tissues with known mTOR pathway-activating variants (Extended Data Fig. 6). In both DNs and BCs, we consistently observed clusters of multi-staged enlarged electron-dense lysosomes and numerous multivesicular bodies that are commonly observed in senescent cells^20,21^. None of the normal-size neighboring neurons displayed these intracellular alterations.

Altogether, these findings establish a direct link among the presence of the pathogenic variants, hyperactivation of the mTOR pathway, the expression of a cellular senescence state and the contribution to epileptiform activity as a specific feature of cytomegalic DNs in FCDII. The absence of cellular senescence hallmarks in control epileptic tissues suggests that seizure recurrence is unlikely to be a contributing factor to this process.

### Cellular senescence in a *Depdc5*^cKO^ mouse model of mTORopathy

We next examined whether cellular senescence occurs in a mouse model of mTORopathy, specifically investigating a neuron-specific *Depdc5* knockout strain (*Depdc5*^cKO^) that was previously generated^22^. DEPDC5 is a member of the GTPase-activating protein (GAP) activity toward Rags 1 complex (GATOR1), a repressor of the amino acid-sensing branch of the mTOR pathway^23^. Loss-of-function mutations in *DEPDC5* leading to the constitutive activation of the mTOR kinase are a frequent cause of genetic focal epilepsies and FCDII (refs. ^24–26^). *Depdc5*^cKO^ mice exhibit cortical mTOR pathway activation and have a shortened lifespan due to fatal seizures occurring around 5 months of age (±1 month)^22^.

**Fig. 3:**
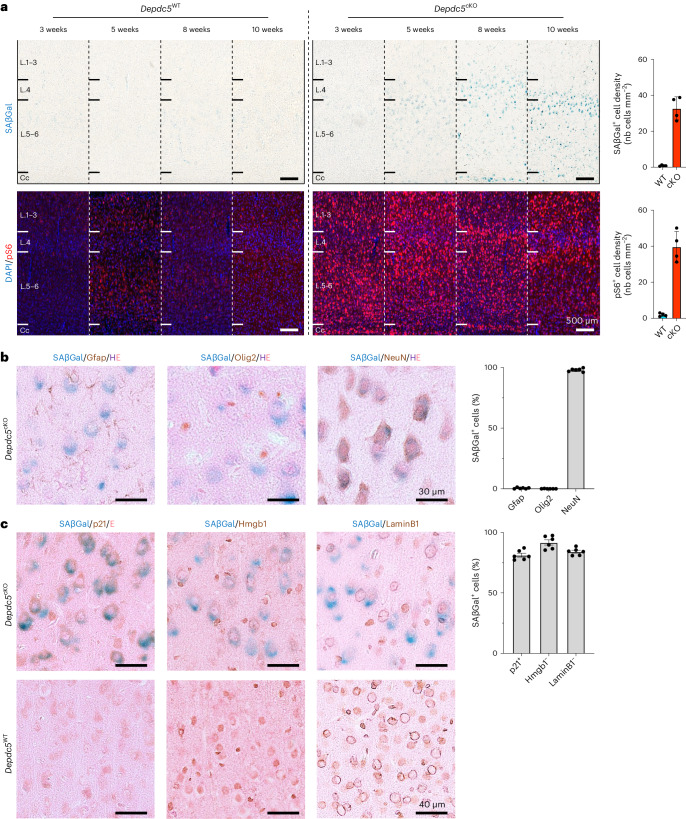
Cellular senescence hallmarks in a mouse model of Depdc5 deficiency. **a**, SAβGal colorimetric assay (top, blue) and pS6 immunostaining (bottom, red) on *Depdc5*^WT^ and *Depdc5*^cKO^ animals from 3 weeks to 10 weeks of age and quantification of the density of SAβGal^+^ and pS6^+^ cells per mm^2^ in 10-week-old *Depdc5*^WT^ (*n* = 4) and *Depdc5*^cKO^ (*n* = 4) mice. Cc, corpus callosum; L.1–3, cortical layers 1 to 3; L.4, cortical layer 4; L.5–6, cortical layers 5 and 6. **b**, SAβGal colorimetric assay (blue) and immunohistochemistry against neural cellular markers (DAB) on *Depdc5*^cKO^ animals at 10 weeks of age. Quantification of the percentage of SAβGal^+^ cells positive for Gfap, Olig2 or NeuN in *n* = 6 samples of 10-week-old *Depdc5*^cKO^ mice (two slices from rostral and caudal regions per *n* = 3 animals). **c**, SAβGal colorimetric assay (blue) and immunohistochemistry against cellular senescence markers (DAB) on *Depdc5*^WT^ and *Depdc5*^cKO^ animals at 10 weeks of age. Quantification of the percentage of SAβGal^+^ cells positive for p21 and negative for nuclear Hmgb1 and LaminB1 in *n* = 6 samples of 10-week-old *Depdc5*^cKO^ mice (two slices from rostral and caudal regions per *n* = 3 animals). Scatter dot plots are presented as mean ± s.e.m. nb, number of. Source data

**Extended Data Fig. 7:**
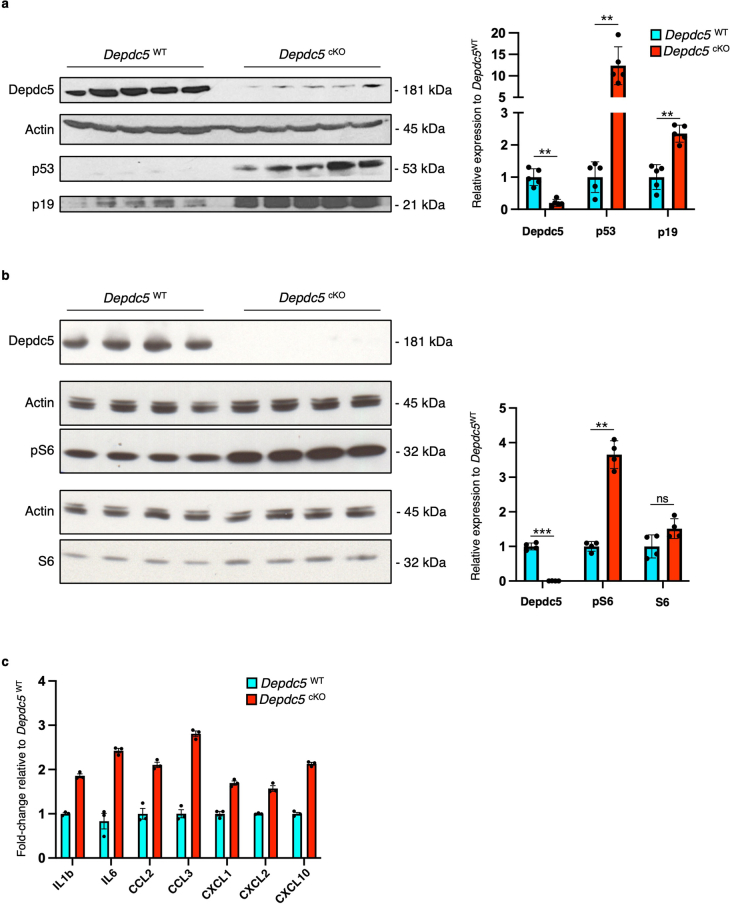
Cellular senescence in a mouse model of Depdc5-deficiency. (**a**) Western blotting against Depdc5, p53 and p19 on n = 5 *Depdc5*^WT^ and n = 5 *Depdc5*^cKO^ 10-weeks old mice (corresponding to 5 biological replicates). Histogram showing the relative expression of Depdc5, p53 and p19 to Actin, normalized to *Depdc5*^WT^. **P = 0.0079; Two-tailed Mann-Whitney test. (**b**) Western blotting against Depdc5, pS6 and total S6 on n = 4 *Depdc5*^WT^ and n = 4 *Depdc5*^cKO^ 10-weeks old mice (corresponding to 4 biological replicates). Histogram showing the relative expression of Depdc5, pS6 and total S6 to Actin, normalized to *Depdc5*^WT^. **P = 0.0286; Two-tailed Mann-Whitney test. (**c**) Histograms showing the quantification of canonical SASP interleukins and cytokines production in n = 3 *Depdc5*^WT^ and n = 3 *Depdc5*^cKO^ brain lysates at 10-weeks of age (averaging 2 technical replicates per animal). Values are normalized to the mean of Depdc5^WT^. Dots indicate all replicates. Scatter dot plots are presented as mean ± SEM. Source data

We first assessed the timing of emergence of both the cellular senescence phenotype and the activation of the mTOR pathway in *Depdc5*^cKO^ animals. For this purpose, we performed temporal series of SAβGal colorimetric assay and pS6 immunofluorescence staining on cortical slices from mice aged 3–10 weeks, before seizure onset, and observed that SAβGal reactivity was detected in *Depdc5*^cKO^ mice from 8 weeks of age. Hyperactivation of the mTOR pathway, observed by increased pS6 levels in *Depdc5*^cKO^ mice, was already detected at 3 weeks of age and was more pronounced than in *Depdc5*^WT^ littermates (Fig. 3a). SAβGal reactivity was never observed in brain sections of *Depdc5*^WT^ littermate mice, indicating that cellular senescence is due to Depdc5 inactivation leading to mTOR activation. Consistent with the neuronal specificity of *Depdc5* knockout in this mouse model, 98 ± 0.5% of SAβGal^+^ cells were co-stained with NeuN (neuronal marker), whereas less than 0.5 ± 0.2% were co-stained with Gfap (astrocyte marker) and Olig2 (oligodendrocyte marker) (Fig. 3b). At the age of 10 weeks, when SAβGal reactivity becomes evident, we measured the levels of p53 and p19, two established senescence markers in mice, through western blot analysis. We found an upregulation of all senescent markers in *Depdc5*^cKO^ brain lysates compared to *Depdc5*^WT^, together with increased phosphorylation levels of S6 protein (Extended Data Fig. 7a,b). We further examined additional senescence markers for cell cycle, nuclear envelope integrity and regulators of the SASP. Immunohistochemistry on *Depdc5*^cKO^ brain sections, together with the SAβGal colorimetric assay, revealed co-occurrence in most of SAβGal^+^ cells of three additional cellular senescence hallmarks: p21 expression and Hmgb1 and LaminB1 nuclear loss (SAβGal^+^p21^+^: 81 ± 1.6%; SAβGal^+^nuclear Hmgb1^−^: 92 ± 2.1%; SAβGal^+^nuclear LaminB1^−^: 84 ± 1.2%; Fig. 3c). In conclusion, cellular senescence emerges in brain cells after mTOR hyperactivation and independently from the occurrence of electro-clinical seizures, which manifested later at approximately 5 months of age in *Depdc5*^cKO^ mice.

A key characteristic of senescent cells is the capacity to secrete pro-inflammatory molecules, including interleukins, cytokines and growth factors, a process known as the SASP^27^. mTOR signaling pathway participates in the regulation of the SASP, both through transcriptional and translational modulation of the expression of at least eight core SASP interleukins and cytokines: IL1b, IL6, IL8, CCL2, CCL20, CXCL1, CXCL2 and CXCL10 (refs. ^15,16,28^). We investigated the production of pro-inflammatory analytes in whole-brain lysates from *Depdc5*^cKO^ and *Depdc5*^WT^ littermates aged 5 weeks and 10 weeks, two timepoints framing the emergence of SAβGal reactivity observed at 8 weeks. We used a multiplex electrochemiluminescent assay to measure 29 analytes, including seven of the eight mTOR-regulated SASP molecules (IL1b, IL6, CCL2, CCL20, CXCL1, CXCL2 and CXCL10). We detected the production of 15 of 29 pro-inflammatory analytes and observed an increase in the production of all seven mTOR-regulated SASP molecules in 10-week-old *Depdc5*^cKO^ compared to *Depdc5*^WT^ animals (Extended Data Fig. 7c and Supplementary Table 3). No differential levels were measured at 5 weeks of age (except CXCL10), consistent with the near absence of cellular senescence hallmarks at that stage (Supplementary Table 3). Therefore, *Depdc5*^cKO^ mice produce SASP molecules detectable in brain lysates from 10 weeks of age, an age at which cellular senescence hallmarks are observable.

### Senolytics reduce seizure frequency in *Mtor*^S2215F^ mice

**Fig. 4:**
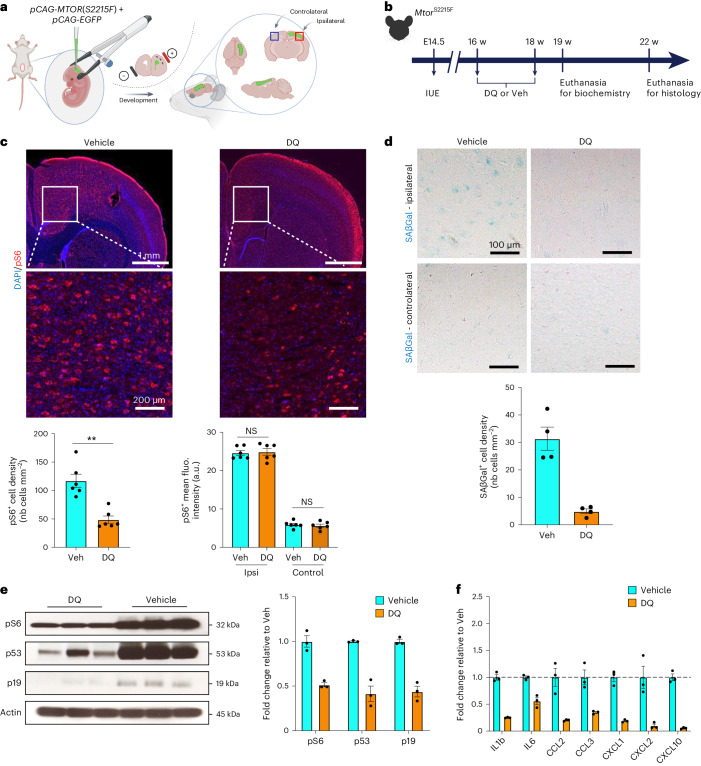
Clearance of senescent cells after DQ administration in *Mtor*^S2215F^ animals. **a**, Experimental design of IUE targeting layer 2/3 pyramidal cell-destined progenitor cells lining the dorsal ventricular zone at E14.5 and subsequent localization of the electroporated area in one hemisphere. **b**, Study design for IUE, histological and biochemical experiments. **c**, Top, representative images of immunofluorescent staining against pS6 (red) and DAPI (blue) on *Mtor*^S2215F^ mice after vehicle or DQ administration. Bottom, quantification of pS6^+^ cell density and pS6^+^ cell mean fluorescence intensity on *n* = 6 vehicle-treated and *n* = 6 DQ-treated *Mtor*^S2215F^ animals (each dot corresponds to one animal). **P = 0.0022 and NS, not significant, two-tailed Mann–Whitney test. **d**, Top, representative bright-field images of SAβGal colorimetric assay (blue) on *Mtor*^S2215F^ animals after vehicle or DQ administration in ipsilateral and controlateral regions. Bottom, quantification of SAβGal^+^ cell density in ipsilateral on *n* = 4 vehicle-treated and *n* = 4 DQ-treated *Mtor*^S2215F^ animals (each dot corresponds to one animal). **e**, Western blot on electroporated cortical brain lysates against pS6, p53 and p19 from *n* = 3 vehicle-treated and *n* = 3 DQ-treated *Mtor*^S2215F^ mice. Histogram showing the relative expression of pS6, p53 and p19 to actin (each dot corresponds to one animal). **f**, Histograms showing the quantification of canonical SASP cytokine production in the same brain lysates as **d** of *n* = 3 vehicle-treated and *n* = 3 DQ-treated *Mtor*^S2215F^ animals (averaging two technical replicates per animal). Values are normalized to the mean of vehicle-treated *Mtor*^S2215F^ animals. Scatter dot plots are presented as mean ± s.e.m. fluo., fluorescence; nb, number of; Veh, vehicle; w, weeks. Source data

**Extended Data Fig. 8:**
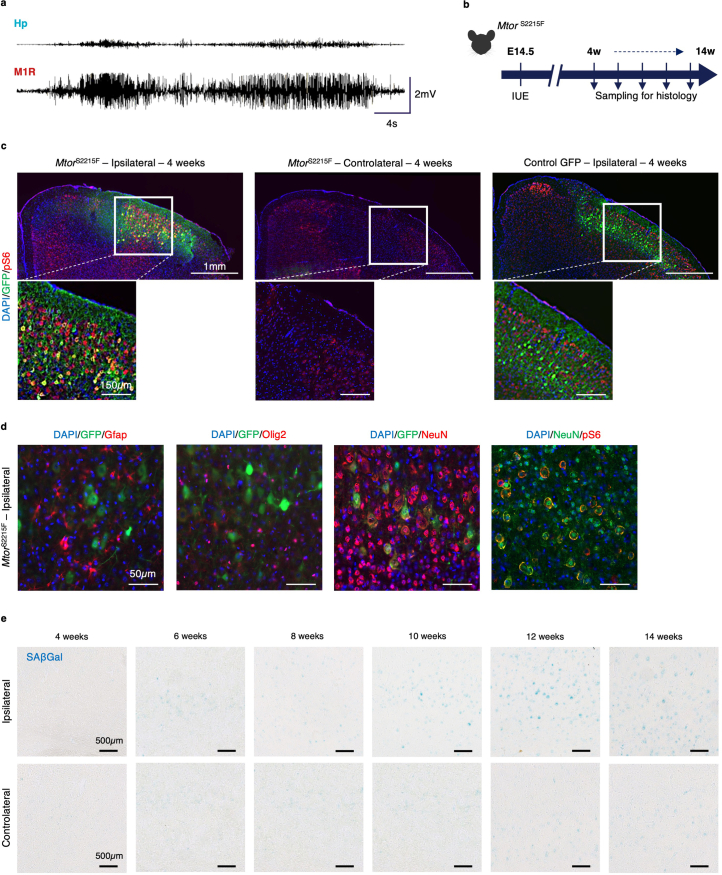
Histological and seizure characteristics of *Mtor*^S2215F^ animals. (**a**) EEG trace of a spontaneous seizure occurring at 6 weeks of age *Mtor*^S2215F^ mouse; ictal activity is mostly seen in the cortex. Hp: hippocampal electrode; M1R: right motor cortex area electrode (**b**) Study design for histology analyses. (**c**) Immunofluorescence against pS6 (red), GFP (green) and DAPI (blue) on *Mtor*^S2215F^ and control mice aged 4 weeks. (**d**) Immunofluorescence against DAPI (blue), GFP or NeuN (green) and neural markers or pS6 (red) on *Mtor*^S2215F^ mice aged 4 weeks. (**e**) SAβGal colorimetric assay (blue) on *Mtor*^S2215F^ mice from 4 to 14 weeks of age in the electroporated area (top, ipsilateral) and corresponding cortical region of the opposite hemisphere (bottom, contralateral). Colorimetric assays were repeated twice and immunostainings were repeated at least three times.

**Extended Data Fig. 9:**
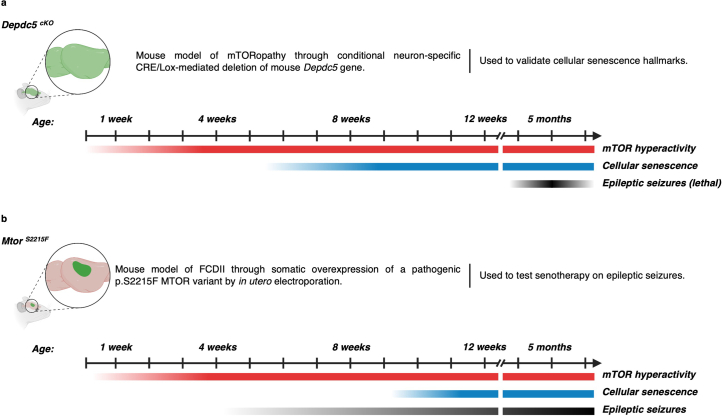
Summary of cellular and behavioral phenotypes in *Depdc5*^cKO^ and *Mtor*^S2215F^ mouse models. (**a**) Description of the Depdc5cKO mouse model of mTORopathy. (**b**) Description of the Mtor^S2215F^ mouse model of FCDII. The timeline of mTOR hyperactivity (red), cellular senescence (blue) and epileptic seizure (black) onsets is represented.

To explore the potential of targeting senescent cells using senolytics—a novel class of molecules that selectively eliminate senescent cells in vitro and in vivo^29,30^—in mitigating the epileptic phenotype, we switched to an in utero electroporation (IUE)-based mouse model. This model expresses the hotspot *MTOR* p.S2215F variant in a mosaic, focal pattern, reflecting brain mosaicism as in patients (*Mtor*^S2215F^) (Fig. 4a). This FCDII mouse model is clinically relevant because it recapitulates key FCDII histopathological hallmarks, including abnormal neuronal migration and the presence of mTOR hyperactive pS6^+^ cytomegalic neurons^31^, and exhibits recurrent spontaneous electro-clinical seizures starting from 6 weeks of age until at least 42 weeks of age (Extended Data Fig. 8a; summary in Extended Data Fig. 9).

We electroporated the *Mtor*^S2215F^ construct in embyonic day (E) 14.5 mouse embryos, targeting radial glia that generate pyramidal neurons of cortical layers 2/3 to model somatic mutations in vivo (Extended Data Fig. 8b–d). We observed SAβGal reactivity in the electroporated cortical area containing mTOR hyperactive (pS6^+^) in mice from 10 weeks of age but not in the contralateral non-electroporated cortical area (Extended Data Fig. 8e). We, therefore, used the *Mto*r^S2215F^ mouse model to test the action of senolytics on reducing senescent cells and ultimately controlling seizures.

We selected the dasatinib and quercetin (DQ) senolytic cocktail previously shown to cross the blood–brain barrier and to clear senescent brain cells in aging mice by transiently blocking senescent cell anti-apoptotic pathways^32,33^. Dasatinib (D) is a pan tyrosine kinase inhibitor, and quercetin (Q) is a natural flavonoid acting on the anti-apoptotic protein Bcl-xL. We administered the DQ cocktail (12 mg kg^−1^ dasatinib and 50 mg kg^−1^ quercetin) or vehicle (DMSO) by oral gavage for 9 days to six *Mtor*^S2215F^ mice in each group at 16 weeks of age (after epilepsy onset) (Fig. 4b). One month after the end of treatment, we quantified the DN-like pS6^+^ cell density within the electroporated area. We observed that DQ treatment significantly reduced the number of pS6^+^ neurons (nearly 70%) compared to the vehicle condition (Fig. 4c). In the remaining non-eliminated DN-like cells, confirmed by Nissl coloration, pS6 levels were unchanged, suggesting that DQ did not lower mTOR activity (Fig. 4c and Supplementary Fig. 1). Consistently, SAβGal assay showed a reduction of about 80% of SAβGal^+^ cytomegalic cells (Fig. 4d). To further confirm the reduction of pS6^+^SAβGal^+^ cells, we microdissected the GFP^+^ electroporated area of DQ-treated (*n* = 3) and vehicle-treated (*n* = 3) *Mtor*^S2215F^ mice 1 week after the end of the treatment. We used western blotting to confirm a decrease of over 50% in pS6, p53 and p19 protein abundance in brain lysates of DQ-treated animals compared to those treated with vehicle, consistent with the partial clearance of senescent mTOR hyperactive cells (Fig. 4e). Brain lysates from the same animals were used to quantify SASP molecule production by electrochemiluminescent assay. Among the eight mTOR-related SASP molecules, the production of the same six found in *Depdc5*^cKO^ animals (IL1b, IL6, CCL2, CXCL1, CXCL2 and CXCL10) was decreased in DQ-treated compared to vehicle-treated animals (Fig. 4f and Supplementary Table 4). In summary, the data demonstrate that DQ senotherapy results in a decrease in the number of mTOR hyperactive cells, reduces the protein expression of cellular senescence markers and lowers the production of SASP molecules in *Mtor*^S2215F^ mice.

**Fig. 5:**
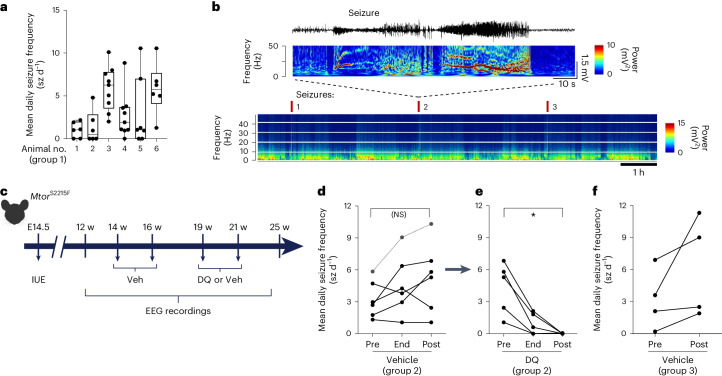
Beneficial effect of DQ administration on epileptic seizures in *Mtor*^S2215F^ animals. **a**, Mean daily seizure frequency in *n* = 6 individual *Mtor*^S2215F^ animals over 6–9 recording sessions on three consecutive days (410–641 h). Data are presented as box plots and have the following values (minimum, 25th percentile, median, 75th percentile, maximum, mean, s.d. and s.e.m.): 1 (0, 0, 1, 2, 2.2, 1, 0.92 and 0.38); 2 (0, 0, 0.48, 2.8, 4.8, 1.3, 1.9 and 0.78), 3 (2, 3.5, 6.3, 7.8, 10, 5.9 and 2.6); 4 (0, 1, 1.9, 3.7, 8.8, 2.7, 2.6 and 0.88); 5 (0, 0, 1, 7, 11, 3, 4.1 and 1.6); and 6 (1.3, 4.1, 5.7, 7.7, 11 and 5.8, 3). **b**, Top, representative EEG trace and corresponding FFT power spectrum over 1 min of recording. Bottom, representative color-coded FFT power spectrum over 7 h of recording showing seizures annotated with red ticks and reflected by sharp increases in frequency and amplitude. The data shown were extracted from one cortical electrode located in the somatosensory S1 cortical area of one non-treated *Mtor*^S2215F^ animal. **c**, Study design for longitudinal in vivo experiments and timepoints of analyses. **d**, Mean daily seizure frequency in *n* = 6 individual *Mtor*^S2215F^ animals over 2 weeks before vehicle administration (‘Pre’), over 48 h after the end of vehicle administration (‘End’) and over 3 weeks after vehicle administration (‘Post’). Statistics: two-tailed Mann–Whitney test. Not significant (NS): *P* = 0.4375. **e**, Mean daily seizure frequency in the same *n* = 5 individual *Mtor*^S2215F^ animals over 3 weeks before DQ administration (‘Pre’), over 48 h after the end of DQ administration (‘End’) and over 4 weeks after DQ administration (‘Post’). One animal (dashed lines) died from seizures during the interphase between vehicle and DQ administration. Statistics: two-tailed Mann–Whitney test. *P = 0.0312. **f**, Mean daily seizure frequency in *n* = 4 *Mtor*^S2215F^ animals over 2 weeks before (‘Pre’) and 2 weeks after (‘Post’) vehicle administration. Each dot corresponds to one animal. Veh, vehicle; w, weeks. Source data

Subsequently, we investigated if lowering pathogenic cell density with acute DQ senotherapy could reduce seizure frequency. We implanted *Mtor*^S2215F^ mice (referred to as group 1, *n* = 6) with cortical electrodes for video electroencephalogram (EEG) recordings from 12 weeks of age. The mean seizure frequency over 4 weeks of video EEG recordings (>230 h) ranged from 1.2 to 7.6 seizures per day (Fig. 5a,b). The variability in seizure frequency observed between animals (seizure frequency variability assessed with *P* = 0.043) may result from the procedure of IUE itself, generating variable numbers and localization of GFP^+^ cells within the targeted cortical region. Next, we assessed the effect of DQ on seizure frequency. To circumvent a putative bias inherent to interindividual variability of daily seizure frequency, we designed a longitudinal study such that each mouse received successively the vehicle and then DQ, in a longitudinal procedure (Fig. 5c). In this second group of *Mtor*^S2215F^ mice (group 2, *n* = 6), implanted with cortical electrodes at 12 weeks of age, we measured the average seizure frequency over a 2-week period before administrating vehicle for 9 days, at the end of treatment, and for 1 month after treatment, using video EEG recordings spanning more than 135 h. Mice had an average of 0.4 and nine seizures daily, similar to seizure frequency in group 1, with no statistical difference before or after vehicle administration (Fig. 5d). In the same group of mice, we then administered DQ for 9 days and assessed the seizure frequency at the end of the treatment and 1 month later (spanning >200 h of video EEG recordings). At the end of the treatment, we observed a significant reduction in the mean daily seizure frequency. One month after DQ treatment, all *Mtor*^S2215F^ mice were seizure free (Fig. 5e). To determine whether this effect might result from vehicle administration alone, we quantified the mean seizure frequency in a third group of *Mtor*^S2215F^ mice (group 3, *n* = 4) over 2 weeks before and 2 weeks after administrating two doses of a vehicle following the same protocol as in group 2 (Fig. 5c). Before vehicle administration, mice exhibited an average of 0.2 to 6.9 seizure episodes daily and, after vehicle administration, between 1.9 and 11.3 seizures daily, indicating that the vehicle has no significant effect on seizure frequency over time (Fig. 5f). Altogether, these results demonstrate that DQ senotherapy can decrease the load of senescent brain cells in vivo and reduce the epileptic seizure burden in *Mtor*^S2215F^ mice.

To assess the potential side effects of DQ, we administrated DQ or vehicle to a group of wild-type (WT) mice (*n* = 6) and monitored body weight and grimace score as means of well-being assessment. Neither a significant variation in body weight nor adverse effects on well-being were observed (Supplementary Fig. 2).

We next evaluated whether DQ could have an anti-epileptic effect by itself using the experimental pentylenetetrazol (PTZ)-induced epilepsy model. DQ or vehicle was administered over 9 days to two groups of WT animals, followed by PTZ injection (40 mg kg^−1^). Seizure severity scores assessed using the Racine scale showed no statistical difference between the DQ or vehicle groups. Moreover, we confirmed the absence of cellular senescence hallmarks (SAβGal reactivity) in PTZ-injected mice (Supplementary Fig. 3). These results suggest that DQ exerts its anti-epileptic effect by specifically targeting senescent cells.

## Discussion

Brain somatic mosaicism is increasingly recognized as a pivotal contributor to neurodevelopmental disorders^34^. FCDII showcases how somatic mutations can disrupt human cortical development and give rise to epilepsy due to a subset of mutated neurons (reviewed in refs. ^5,6^). Here we provide compelling evidence of the presence of a cellular senescence signature in pathological cells of mTOR-related FCDII, which contribute to generating epileptiform activity in acute FCDII cortical slices. We further show that administration of DQ senolytic cocktail reduces the load of senescent cells and seizure frequency in an Mtor^S2215F^ FCDII preclinical mouse model.

In human FCDII tissue, we discovered that DNs and BCs displayed a senescence phenotype as assessed by a multi-layer approach, including SAβGal reactivity, activation of p53 and p16 pathways, loss of nuclear envelop integrity, high lysosomal content and acquisition of an SASP. Likewise, cellular senescence hallmarks were also present in two mouse models of mTORopathy (*Depdc5*^cKO^) and FCDII (*Mtor*^S2215F^). Altogether, these findings support the rationale of using the combination of dasatinib and quercetin (DQ cocktail), which has been shown to decrease the senescent cell burden and the SASP in vitro, in mouse models of age-related diseases^32,35^ and glioblastoma^36^ and in human tissues^29,32,37,38^. In the present study, administration of the DQ senolytic cocktail to a preclinical FCDII *Mtor*^S2215F^ mouse model reduced the pathological cell load and seizure frequency. Absence of seizure recurrence up to 1 month after drug discontinuation suggests that, in contrast to rapamycin administration in other mTORopathy models^13,39,40^, the DQ cocktail provides a stable improvement of the epileptic phenotype by eliminating seizure-causing cells.

Cellular senescence is commonly associated with both normal aging and age-related conditions^33,35,41,42^. Hence, the discovery of premature cellular senescence-like features in pediatric brain epileptic tissues is particularly noteworthy. The developmental emergence of the senescence phenotype is unknown. In humans, the difficulty in accessing fetal cortical FCDII tissue hinders the ability to determine whether cellular senescence arises during the proliferative phase of corticogenesis in progenitors or postnatally in post-mitotic neurons. However, in the two mouse models examined, cellular senescence occurred postnatally, around 8–10 weeks of age.

An association between mTOR signaling and cellular senescence was previously reported in vivo in the context of aging^15,16^. Moreover, in TSC, loss-of-function mutations in *TSC1/TSC2*, which lead to increased mTOR activity, also induce premature cellular senescence in murine *Tsc2*^−/−^ fibroblasts^43^, and mTORC1 signaling coordinates the senescence program through the complementary action of 4EBP and S6K that selectively control p53/p21 and p16 in *TSC1*^−/−^ cultured fibroblasts^44^. Our findings in two mouse models (*Depdc5*^cKO^ and *Mtor*^S2215F^) indicate that the activation of mTOR signaling is followed by the onset of cellular senescence (summarized in Extended Data Fig. 9).

By integrating electrophysiological, histopathological and genetic data from cortical slices obtained from two patients with FCDII, we showed that senescent-like DNs are preferentially located in the cortical area that displays spontaneous interictal-like activity. How a few scattered mutated brain cells drive the network hyperexcitability in the focal dysplastic cortex is still controversial. Two possible scenarios for network hyperexcitability include intrinsic hyperexcitability of mutated DNs and/or indirect effects on interneurons by the projections of mutated pyramidal cells. Previous studies indicated the important role of DNs in the generation and propagation of epileptic discharges^45,46^, supported by findings that intrinsic epileptogenicity is maximal in the dysplastic sulcus with high DN density^47^, and a higher mutation load in regions with frequent, high-voltage spike waves and fast ripples^48^. Intracerebral EEG and histology co-registration also support the contribution of DNs to interictal spikes and fast gamma activity^49^. Surgical removal of the DN-containing area in patients with FCDII often leads to seizures, emphasizing intrinsic epileptogenicity being confined to the dysplastic lesion. Consistent with these findings, targeted electrical silencing of mTOR hyperactivated cells prevents seizures in a mouse model of FCDII (ref. ^50^). Supporting a scenario in which altered cortical inhibitory circuitry contributes to network hyperexcitability, some studies reported altered GABA-mediated synaptic inhibition in various types of cortical dysplasias^51–53^. Further research will, thus, be needed to establish the extent to which cell-autonomous and non-cell-autonomous processes interact to give rise to the epileptic phenotype. In human FCDII, it is unlikely that cellular senescence is a consequence of neuronal hyperactivity because both ‘hyperexcitable’ DNs and ‘electrically silent’ BCs display a senescence phenotype. In the two mouse models, the onset of cellular senescence and seizures differed, suggesting that senescence by itself does not cause and is not directly caused by seizures (summarized in Extended Data Fig. 9).

This study has some limitations. First, although we used cellular senescence as a targetable biomarker, the putative mechanistic role of neuronal senescence in epileptogenesis remains largely unknown. Cellular senescence in mitotic cells is generally recognized as a response to stress, producing specific signals that induce inflammation and local tissue dysfunction. However, the mechanisms governing senescence in post-mitotic cells, such as neurons, are poorly understood^19^. Second, the mechanisms by which senolytics induce brain cell clearance and potential compensatory processes remain uncertain. Third, senolytics might elicit off-target effects, potentially affecting physiological senescent cell populations elsewhere in the body. Cellular senescence can play beneficial roles during neurodevelopment, underscoring the need for careful consideration when proposing senolytics as therapeutic interventions, particularly in pediatric cases^37^. However, selectively targeting these low-abundant pathogenic cells for partial removal could offer a less invasive alternative compared to the surgical resection of the whole epileptogenic zone.

Most patients with FCDII do not respond to conventional anti-seizure medication; a clinical trial with sirolimus, an mTOR inhibitor, showed no significant seizure reduction^54^. This study highlights the therapeutic potential of repurposing senolytic drugs that efficiently cross the blood–brain barrier and induce apoptosis in senescent cells in the clinical management of mTOR-related epilepsy. Senolytics have demonstrated promising therapeutical potential in several diseases, as outlined by many ongoing clinical trials (reviewed in ref. ^29^). Our study innovatively repurposes the DQ senolytic cocktail for a mosaic neurodevelopmental disorder, where senescent cells are present in low abundance due to somatic mosaicism, paving the way for ultra-precision therapy—that is, targeting mutated cells.

In conclusion, this novel therapeutic approach brings three original aspects. First is the possibility of using precision medicine to selectively target the small subset of pathogenic mutated cells, making it particularly suitable for mosaic disorders. Second, this strategy is potentially applicable to all FCDII cases, regardless of the mutated gene. Finally, we anticipate that senolytics will offer a lasting beneficial effect without the immediate recurrence of seizures, as they appear to permanently reduce the number of abnormal cells instead of transiently modulating their activity. Our study introduces a disruptive therapeutic approach distinct from conventional treatments with chronic anti-epileptic drugs, potentially extending to other mTORopathies, such as TSC or mosaic PIK3CA-related overgrowth syndromes, among others.

## Methods

### Patients, neuropathology and genetic testing

Brain specimens from 37 patients operated for drug-resistant epilepsy (aged from 3 months to 16 years) at the Rothschild Foundation Hospital in Paris, France, between 2016 and 2020 were investigated for molecular and cellular studies. The cohort consisted of cases with FCDII or hemimegalencephaly (HME) (*n* = 24) and epilepsy surgical cases used as controls with a neuropathological diagnosis of FCDI (*n* = 5) or mild malformation of cortical development (mMCD; *n* = 8). Non-essential brain tissues for neuropathological diagnostic purposes were attributed to research by the neurosurgeon in agreement with the neuropathologist. All specimens were immediately frozen in liquid nitrogen except four FCDII brain specimens that were fixed immediately after surgery for electron microscopy. Post hoc genetic analysis on frozen bulk tissue was performed by targeted deep sequencing using a gene panel consisting of approximately 50 genes involved in mTOR signaling and focal cortical malformations, as previously published^8^. In addition, two FCDII samples (patient ID 8 and patient ID 9) were provided by the Sainte-Anne and Lariboisière Hospitals and were kept oxygenated for ex vivo MEA recordings after surgery. Neuropathological diagnosis was made according to the classification of the Diagnostic Methods Commission of the International League Against Epilepsy^55,56^.

### MEA recordings on human cortical slices

Cortical specimens collected in the surgery room were immediately transported to the laboratory within 15 min in ice-cold (0–4 °C) oxygenated solution (O_2_/CO_2_ 95/5%), containing (in mM): *N*-methyl-d-glucamine 93, KCl 2.5, NaH_2_PO_4_ 1.2, NaHCO_3_ 30, HEPES 20, D-glucose 20, ascorbic acid 5, sodium pyruvate 3, MgSO_4_ 10 and CaCl_2_ 0.5 (300–310 mOsm, pH 7.4). Transverse 400-μm-thick cortical slices were prepared in the same solution using a vibratome (HM650V, Microm). They were maintained at 37 °C in an interface chamber containing artificial cerebrospinal fluid (ACSF) composed of (in mM): D-glucose 10, KCl 3.5, NaHCO_3_ 26, NaH_2_PO_4_ 1.25, NaCl 126, CaCl_2_ 1.6 and MgCl_2_ 1.2 (290 mOsm), equilibrated with 5% CO_2_ in 95% O_2_. MEA recordings were performed using an MEA2100 station (MultiChannelSystems) equipped with a 120-microelectrode array chamber (10 × 12 layout, 30 µm TiN electrodes spaced 1,000 µm vertical and 1,500 µm horizontal). Slices were maintained in the recording chamber using a homemade platinum/nylon harp and perfused with pre-warmed (37 °C) oxygenated ACSF at a rate of 6 ml min^−1^. Slices were imaged using a video microscope table (MEA-VMT1, MultiChannelSystems) to register the location of electrodes with respect to the slice. Extracellular signals were acquired at a sampling rate of 10 kHz and filtered with a low-pass Bessel filter (order 2; 40 Hz) or high-pass Bessel filter (order 2; 100 Hz) using Multi Channel Experimenter (MultiChannelSystems, version 2.20). Analyses were performed offline using homemade software (MATLAB, version R2018b). The semi-automated IILD and MUA detection was performed according to a standard procedure previously reported^57^. In brief, for IILD detection, the signal was denoised, filtered in the 1–40-Hz range, squared and then normalized over the entire recording. IILD detection was then semi-automatic, using a user-defined threshold. For MUA, a similar procedure was used on signal high-pass filtered above 250 Hz. SigmaPlot 13.1 (SPSS) was used for statistical analysis and graph generation.

### Immunofluorescence on human acute cortical slices

Immediately after MEA recordings, 400-µm-thick slices were fixed in 4% paraformaldehyde (PFA) for 12 h before transfer in PBS. Slices were blocked in PBS + 2% Triton X-100 + 2% BSA + 10% normal donkey serum for 48 h on an orbital shaker at room temperature. Slices were then incubated in PBS + 1% Triton X-100 + 1% normal donkey serum with appropriate primary antibody for 48 h on an orbital shaker at 4 °C. After 3 × 30-min rinsing in PBS, slices were incubated in PBS + 0.5% Triton X-100 with appropriate secondary antibody and 0.1 mg ml^−1^ DAPI for 24 h on an orbital shaker at 4 °C. Slices were then rinsed in PB 0.1 M for 24 h on an orbital shaker at room temperature. Finally, slices were subjected for 24 h on an orbital shaker at room temperature to tissue clarification in RapiClear (SUNJIN Lab, RapiClear 1.49), mounted on homemade microscope slide mounts and imaged using a Nikon A1R HD25 confocal microscope with a ×10 glycerol objective in resonant scan mode. z-stacks per 0.16-mm^2^ field of acquisition were acquired with a 10-µm step over the 400-µm thickness at the three color-coded dashed areas in the tissue. Between 10 and 170 DNs were counted per area.

### Orientation of cortical slices for correlation between MEA and immunostainings

After MEA recordings, 400-µm-thick slices were individually placed in 12-well plates, fixed in 4% PFA and immunostained in the same well to preserve tissue integrity. Superimposition of images of slices taken during MEA recordings and after immunostaining was performed based on selective anatomical markers visually identified from the images. Finally, high-resolution confocal imaging was performed in the selected outlined area.

### SaβGal colorimetric assay and immunohistochemistry

#### Human tissue

SaβGal colorimetric assay was performed following the manufacturer’s protocol (Cell Signaling Technology, 9860) with an incubation period of 12 h. All flash-frozen human brain slices (cases and controls) were processed together in the exact same experimental conditions, such as pH, temperature, humidity and incubation time—critical parameters that may influence SaβGal reactivity. Cortical sections were then fixed for 15 min in 4% PFA before washing in PB 0.1 M and immediate subsequent immunohistochemistry. For immunohistochemistry, samples were incubated in 1% H_2_O_2_ for 5 min, rinsed three times in PBS–Tween 20 0.05% and blocked in PBS–Tween 20 0.05% + 10% normal goat serum + 0.2% Triton X-100 for 30 min. Slides were then incubated in appropriate primary antibody diluted in blocking solution overnight at 4 °C. The next day, samples were rinsed three times in PBS–Tween 20 0.05% and incubated in appropriate HRP-coupled secondary antibody diluted in blocking solution for 1 h at room temperature. Slides were then rinsed three times in PBS–Tween 0.05% before incubation in amplification ABC vector kit (Vector Laboratories, PK6100). Samples were then rinsed three times in PBS–Tween 0.05% and dipped in ddH_2_O, and revelation was performed using a DAB kit (Vector Laboratories, SK4100) before standard counterstaining with hematoxylin and eosin. Samples were finally dehydrated in ethanol solutions before mounting in xylene and automatic scanning using a NanoXoomer slide scanner (Hamamatsu). Images were visualized using NDP.view 2 software (Hamamatsu). The following antibodies were used: p53 (1:300, DAKO, M7001, mouse), p16 (1:200, Abcam, 108349, rabbit), pS6 S240/244 (1:1,000, Cell Signaling Technology, 5364, rabbit), SMI311R (1:400, BioLegend, 837801, mouse), VIM (1:400, DAKO, M0725, mouse), p21 (1:200, Abcam, ab188224, rabbit), Hmgb1 (1:100, Cell Signaling Technology, 6893, rabbit), LaminB1 (1:100, Cell Signaling Technology, 17416, rabbit), NeuN (1:500, Merck, MAB377, mouse), Olig2 (1:200, Abcam, ab109186, rabbit | 1:100, Merck, MABN50, mouse) and Gfap (1:300, LifeTechnologies, MA515086, mouse).

#### Mouse tissue

Mouse brain slices were processed as human brain slices only with a shorter incubation period of 6 h. Quantifications of SaβGal^+^ cells were performed semi-automatically on Fiji software (ImageJ2, version 2.9.0/1.53t). In brief, RGB images were used to set a detection threshold for blue pixels on WT brain tissues. The threshold was then applied to images of vehicle-treated or DQ-treated animals and individually saved to ROI Manager. Automatic counting of objects greater than 25 px^2^ was performed and used as a proxy of the number of SaβGal^+^ cells in the different considered areas. For immunostainings, mounted slices were permeabilized for 1 h in PBS + 0.2% Triton X-100 + 5% BSA and then incubated in PBS + 1% BSA with primary antibody against pS6 S240/244 (1:1,000, Cell Signaling Technology, 5364, rabbit) overnight at 4 °C. Slides were then rinsed in PBS and incubated in PBS with secondary antibody anti-rabbit Alexa Fluor 555 (Thermo Fisher Scientific, A27039, goat) for 1 h at room temperature, before washing and 0.1 mg ml^−1^ DAPI incubation for 10 s. Quantification of pS6^+^ cells was performed semi-automatically on Fiji software. Eight-bit images were used to set a user-based fluorescence detection threshold, converted into binary files and subjected to watershed. Automatic counting of objects greater than 25 px^2^ was performed to quantify the density of pS6^+^ cells (that is, the number of objects per field of view).

#### Quantifications

For SAβGal colorimetric assay and DAB co-stainings on human tissue, between *n* = 42 and *n* = 310 SAβGal^+^ cells were manually counted on a 1-cm^2^ region of interest (ROI) per sample on *n* = 5 FCDII samples. For SAβGal colorimetric assay and DAB co-stainings on mouse tissue, between *n* = 359 and *n* = 405 SAβGal^+^ cells were manually counted on two 0.5-mm^2^ somatosensory cortical column ROIs (one rostral and one caudal) per animal on *n* = 3 animals (*n* = 6 samples in total). For fluorescent stainings on human tissue, cellular density (pS6^+^NeuN^+^ DNs and pS6^+^NeuN^−^ BCs) was semi-automatically quantified using the Fiji Analyze Particle module on 2.5-mm^2^ ROIs per sample. For fluorescent stainings on mouse tissue, cellular density and mean fluorescence intensity (pS6^+^ electroporated neurons) were semi-automatically quantified using the Fiji Analyze Particle module on 1-mm^2^ ROIs. For SAβGal colorimetric assay quantification, cellular density was semi-automatically quantified using the Fiji Analyze Particle module on 0.5-mm^2^ ROIs on *n* = 4 animals.

### Electron microscopy on FCDII samples

Samples (*n* = 6) were immediately fixed in the operating room after surgical removal in 2% glutaraldehyde + 2% PFA + 2 mM CaCl_2_ in 0.1 M sodium cacodylate buffer, pH 7.4, for 1 h at room temperature. Tissues were post-fixed with 1% osmium tetroxide in water for 1 h at room temperature, rinsed three times with water and contrasted ‘en bloc’ for 1 h at room temperature with 2% aqueous uranyl acetate. Small pieces (1 mm^3^) of gray matter were dissected and progressively dehydrated in 50%, 70%, 80%, 90% and 100% ethanol solution (10 min each). Final dehydration was performed twice in 100% acetone for 20 min. Infiltration with an epoxy resin (EMbed 812) was performed in two steps: one night at 4 °C in a 1:1 mixture of Epon and acetone in an airtight container and twice for 1 h at room temperature in freshly prepared resin. Finally, samples were placed in molds with fresh resin. Polymerization was performed at 56 °C for 48 h in a dry oven. Blocks were cut with a Leica UC7 ultramicrotome. Semi-thin sections (0.5 μm thick) were stained with 1% toluidine blue in 1% borax, allowing identification of DNs and BCs. Ultra-thin sections (70 nm thick) were contrasted with Reynold’s lead citrate and observed with a Hitachi HT7700 electron microscope operating at 70 kV. Pictures were taken with an AMT41B camera.

### Laser capture microdissection and ddPCR

Laser capture microdissection was performed in two FCDII/HME tissues with pathogenic variants in *MTOR* and *PIK3CA* (ID 3 and ID 13) using a Leica LMD7000 system on 20-µm frozen brain sections mounted on PEN-membrane slides after SAβGal colorimetric assay and immunohistochemistry against pS6. Pools of *n* = 200 double-positive SAβGal^+^pS6^+^ enlarged cells (soma diameter >25 µm) were microdissected and collected in AdhesiveCap 500 Opaque tubes (Zeiss) for DNA extraction. ddPCR was performed as previously described^58^ using specific probes to detect variants *MTOR*:p.T1977K and *PIK3CA*:p.R1047H.

### Mouse models

Two mouse models were used in this study; both males and females were used unless otherwise specified; and all animals were used at adult age (from postnatal day (P) 28). All mice were kept and bred under controlled conditions with a 12-h/12-h light/dark cycle, 45–65% humidity, a temperature of 22 °C as well as food and water ad libitum. All efforts were made to minimize the suffering and number of animals used in this study.

*Depdc5*^cKO^ strains (on a C57BL6/J background, Janvier Labs) were previously reported^22^. *Depdc5*^flox/flox^ mice were generated by flanking exons 1–3 with loxP sites by genOway, a fee-for-service external company. Mice were crossed with Synapsin1-Cre mice (B6.Cg-Tg(Syn1-cre)671Jxm/J, no. 003966, The Jackson Laboratory) to obtain *Depdc5*^flox/flox^;Syn-Cre^+/−^ animals named *Depdc5*^cKO^ and *Depdc5*^flox/flox^;Syn-Cre^−/−^ animals named *Depdc5*^WT^.

For the *Mtor*^S2215F^ model, *Mtor*^S2215F^ mice were generated by IUE at E14.5 in Swiss/CD1 embryos (Janvier Labs) as in ref. ^59^. DNA solution contained 0.5 mg ml^−1^ pCAG-EGFP and 2.5 mg ml^−1^ pCAGIG-mTOR (p.S2215F) (kindly provided by Alfonso Represaʼs team at INMED, Marseille), a plasmid encoding the recurrent *MTOR*: p.S2215F variant found in patients with FCDII. At birth, pups were selected based on GFP fluorescence in the head visualized under a microscope.

### Brain lysate preparation

Animals were killed by beheading. Whole brains of Depdc5^cKO^ mice (*n* = 5) and control littermates (*n* = 5) were dissected out of the skull and immediately placed in a 2-ml microcentrifuge tube dropped in liquid nitrogen. For *Mtor*^S2215F^ models, GFP^+^ cortical regions were scooped out of the brain, placed in a 2-ml microcentrifuge tube and dropped in liquid nitrogen. Lysates were prepared by transferring half of the brain samples in tubes containing FastPrep homogenizer beads with 200 µl of ice-cold lysis buffer (Cell Signaling Technology, 9803) complemented with anti-phosphatase and anti-protease. Homogenization was performed using a FastPrep homogenizer. Homogenate was transferred without beads to a new 1.5-ml microcentrifuge tube and centrifuged at 10,000*g* for 10 min at 4 °C, and supernatant was collected as final solution and stored at −80 °C.

### Western blotting

Total protein concentrations were quantified using a BCA Protein Assay Kit on an automated plate reader (SpectraMax, Molecular Devices). Then, 50 µg of proteins per sample was separated on 4–12% Bis-Tris gel and transferred to a nitrocellulose membrane. After Ponceau coloration to visualize protein bands, membranes were cut according to the targeted protein molecular weights, blocked for 1 h in PBS–Tween 20 0.05% + 5% BSA before incubation in appropriate primary antibody diluted in blocking buffer overnight at 4 °C. Membranes were then incubated in appropriate HRP-coupled secondary antibody for 2 h. Membranes were then rinsed three times and incubated for 5 min in an ECL kit, and revelation was performed on autoradiographic films. Densitometry on Fiji was performed to quantify the expression of targeted proteins normalized to actin. The following primary antibodies were used: Depdc5 (1:250, Abcam, ab185565, rabbit); p53 (1:300, DAKO, M7001, mouse); p19 (1:2, CNIO, rat); actin (1:1,000, Merck, A2066, rabbit); pS6 S240/244 (1:2,000, Cell Signaling Technology, 5364, rabbit); and total ribosomal protein S6 (1:1,000, Cell Signaling Technology, 2317S, mouse). Secondary HRP antibodies from Cell Signaling Technology were used at 1:2,000 (anti-mouse, 7076; anti-rabbit, 7074; and anti-rat, 7077). Biological replicates (3–5 mice for each genotype or condition) were used.

### Multiplex immunoassays

The assay was performed following the manufacturer’s protocol (Meso Scale Discovery (MSD), V-Plex Mouse Cytokine 29-Plex Kit). Brain lysates (250 µg) and neuronal conditioned medium (50 µl) were used per well. Every measure was done in duplicate. *n* = 3 brain lysates per genotype per age were used (with two technical replicates each time), and *n* = 6 conditioned medium per genotype were used. Plates were read on an MSD QuickPlex.

### Mouse surgery and intracranial electrode implantation

*Mtor*^S2215F^ mice (*n* = 16, groups 1, 2 and 3) dedicated for video EEG experiments were selected based on visual confirmation of handling-induced behavioral seizures before intracranial electrode implantation. Mice aged 2 months were administered 0.1 mg kg^−1^ buprenorphine 30 min before being anesthetized with 2% isoflurane and placed in a stereotaxic frame. As previously described^59^, enamel-coated stainless steel electrodes were implanted on the left and right primary motor cortex (M1: AP, 2.2 mm; MD, 2.2 mm), on the right and left lateral parietal association cortex (LPta: AP, −1.8 mm; MD, −1.2 mm) and a common reference in the cerebellum. All coordinates were derived and adjusted from the Paxinos and Watson mice brain atlas^60^.

### Video EEG recordings and analyses

*Mtor*^S2215F^ mice (*n* = 12) were placed under freely moving conditions and connected to an A.D.C. amplifier (BRAINBOX EEG-1166), part of an EEG video acquisition system (DeltaMed, Natus). EEG signals were acquired at 2,048 Hz and band-pass filtered between 0.5 Hz and 70 Hz. The video was synchronized to the electrophysiological signal and recorded at 25 frames per second. *Mtor*^S2215F^ animals were recorded from 3–6 months of age for 72 continuous hours weekly. Analysis of EEG recordings was manually performed by an experimenter blinded to the drug administration protocol. Fast Fourier transform (FFT) analyses were achieved using the Gabor function running in MATLAB (MathWorks, version R2015b) and used to semi-automatically identify ictal events that were then confirmed visually based on pattern criteria used for human patients with epilepsy. Morlet wavelets were computed in the 1–50-Hz frequency.

### Senolytics administration protocol

The typical scheme of drug administration is oral gavage of vehicle (DMSO) or senolytic DQ agents for four consecutive days, followed by 2 days of intermission and five additional days as previously described^32^. Stock solutions were prepared in DMSO 1 day before protocol initiation, aliquoted accordingly and stored at −20 °C as follows: 100 mg ml^−1^ dasatinib (Merck, CDS023389) and 60 mg ml^−1^ quercetin (Merck, PHR1488). The final solution was prepared immediately before gavage at a concentration of 12 mg kg^−1^ dasatinib and 50 mg kg^−1^ quercetin (or equivalent volume of DMSO for preparation of vehicle) with 30% PEG300 and 5% Tween 20 in ddH_2_O. Only one animal died during the protocol between phases of vehicle and DQ administration in one experimental design, likely due to failure in proper gavage procedure (outlined in red on Fig. 5d).

### Animal well-being monitoring

The mouse grimace scale, as outlined by the National Center for the Replacement Refinement and Reduction of Animals in Research^61^, was used for assessing pain in mice as a proxy of well-being after DQ gavage. In brief, a score of 0 (no presence), 1 (moderate presence) and 2 (obvious presence) is given for the observation of five typical facial expressions used as proxies: orbital tightening, nose bulge, cheek bulge, ear position and whisker change. A score of 0 was assigned to each of the five features for both the DQ-treated and the vehicle-treated group of mice, before, during and at the end of the treatment. In parallel, animal well-being was assessed by monitoring body weight every day before the oral gavage procedure.

### PTZ administration and seizure severity assessment

We used the PTZ-induced experimental model of epilepsy^62^. PTZ (Merck, P6500) was dissolved in 0.9% saline at 20 mg ml^−1^. Swiss/CD1 mice (Janvier Labs) received oral administration of the vehicle or DQ for 9 days (as described above) and received, on the 9th day, a single dose of 40 mg ml^−1^ PTZ. Seizures had a rapid onset (average <60 s), spontaneously resolved, were never lethal and were graded by an observer blinded to the experimental condition based on the following modified Racine scale: score 1, sudden arrest; score 2, head twitches and myoclonic jerks; score 3, repetitive forelimb clonus; score 4, generalized tonic-clonic convulsions with loss of righting reflex; and score 5, lethal seizure.

### Statistics

All scatter dot plots with bars are presented as mean ± s.e.m. All statistical analyses were performed in GraphPad Prism 8 (GraphPad Software; Prism 10 for macOS, version 10.1.1 (270)). All statistical analyses were two-tailed. Exact *P* values are provided when possible. Only *P* values lower than 0.05 were considered statistically significant.

### Study approval

For human data, the study protocol received approval by the ethics committee of CPP Île-de-France II (no. ID-RCB/EUDRACT-2015-A00671-48) and the INSERM Ethics Review Committee (no. 22-879) by the INSERM Institutional Review Board (IRB00003888, IORG0003254 and FWA00005831). This study is registered on ClinicalTrials.gov (NCT02890641). Informed and written consent was obtained from all participants (or their parents on their behalf).

For mouse data, the study protocols received approval from the French Ministry of Research (APAFIS 26557, 37296 and 40207). All efforts were made to minimize the suffering and number of animals used in this study.

### Statistics and reproducibility

No statistical methods were used to predetermine sample size. For experiments with quantitative measurements, sufficient numbers of samples were used to derive non-parametric statistical tests. No data were excluded from the analyses. Experimenters were blinded to allocation during experiments and outcome assessment regarding the genotype or the drug (DQ versus vehicle). For in vivo experiments, attribution to experimental groups was randomized. Colorimetric assays and immunostainings on human frozen tissue and mouse tissue were repeated at least three times. Western blots were repeated at least two times. MEA recordings and histology stainings in Fig. 1 could be performed only once on each slice (four slices in patient 8 and two slices in patient 9). Electron microscopy imaging was repeated at least three times (Extended Data Fig. 6). Statistical tests used are non-parametric two-tailed Mann–Whitney tests because no a priori exists regarding the directionality of variation, and no distribution to meet required assumption of normality is required.

### Reporting summary

Further information on research design is available in the Nature Portfolio Reporting Summary linked to this article.

## Online content

Any methods, additional references, Nature Portfolio reporting summaries, source data, extended data, supplementary information, acknowledgements, peer review information; details of author contributions and competing interests; and statements of data and code availability are available at 10.1038/s41593-024-01634-2.

### Supplementary information

Supplementary Information Supplementary Figs. 1–3 and Tables 1–4.

Reporting Summary

### Source data

Source Data Figs. 1–5 and Source Data Extended Data Figs. 1 and 7 Tab Fig. 1a—GitHub link to custom analysis pipelines. Tab Fig. 1c—Quantified data of MEA and statistical tests. Tab Fig. 1e—Quantified data of cell density. Tab Fig. 2a—Quantified data of co-staining (senescence markers). Tab Fig. 2b—Quantified data of co-staining (pathological cell markers). Tab Fig. 2c—Quantified data of co-staining (mTOR activation). Tab Fig. 2d—Quantified VAF in *n* = 2 tissues. Tab Fig. 3a—Quantified data of pS6^+^SAβGal^+^ cell densities. Tab Fig. 3b—Quantified data of co-staining (cell markers). Tab Fig. 3c—Quantified data of co-staining (senescence markers). Tab Fig. 4c—Quantified data of pS6 cell density/mean fluorescence intensity and statistical tests. Tab Fig. 4d—Quantified data of SAβGal cell density. Tab Fig. 4e—Quantified western blot data and uncropped gels. Tab Fig. 4f—Quantified data of SASP analytes assay. Tab Fig. 5a—Quantified data of seizure frequency and statistics. Tab Fig. 5d,e—Quantified data of seizure frequency and statistics. Tab Fig. 5f—Quantified data of seizure frequency. Tab Extended Fig. 1 —Quantified data of cell density. Tab Extended Fig. 7a—Quantified western blot data and uncropped gels. Tab Extended Fig. 7b—Quantified western blot data and uncropped gels. Tab Extended Fig. 7c—Quantified data of SASP analytes assay.

## Data Availability

All data supporting the findings of this study are available in the article and its Supplementary Information. Raw data from electrophysiological recordings are available from the authors upon reasonable request. All materials used in this study are available from the authors upon reasonable request. Given the sensitive nature of human post-surgical samples, original material can be made available from the authors upon request unless entirely used by the time of request, with the exception of original materials from patients ID 8 and 9 that have been already used entirely for the purpose of this study. Source data are provided with this paper.

## Code availability

In vitro electrophysiological data were analyzed offline using custom algorithms written in MATLAB (MathWorks) and are available on GitHub at https://github.com/jcponcer/PoncerLab/tree/740189ac6b202c2182daad7730f4dbfb1d80118e/matlab_interictal_detection.
