# Supplementary material for: Targeting pathological cells with senolytic drugs reduces seizures in neurodevelopmental mTOR-related epilepsy
Source: Nat Neurosci. 2024 May 6;27(6):1125–36. doi: 10.1038/s41593-024-01634-2 (PMC11156583; doi:10.1038/s41593-024-01634-2)
Supplement: Supplementary file 1 — Supplementary Figs. 1–3 and Tables 1–4. [file 41593_2024_1634_MOESM1_ESM.pdf]

nature neuroscience

Article

https://doi.org/10.1038/s41593-024-01634-2

Targeting pathological cells with senolytic drugs reduces seizures in neurodevelopmental mTOR-related epilepsy

In the format provided by the authors and unedited

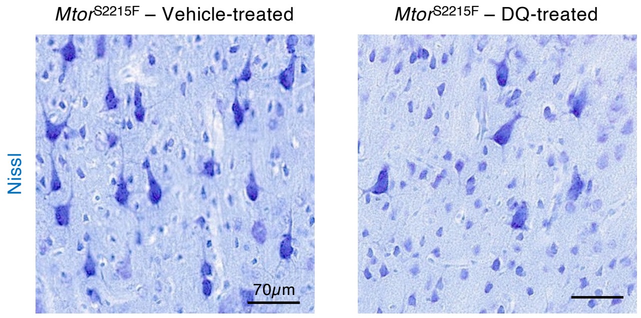

Supplementary Fig. 1. Histological Nissl coloration of cortical slices from DQ-treated and Vehicle-treated *Mtor*^{S2215F} mice. Coloration was repeated three times on n=3 animals.

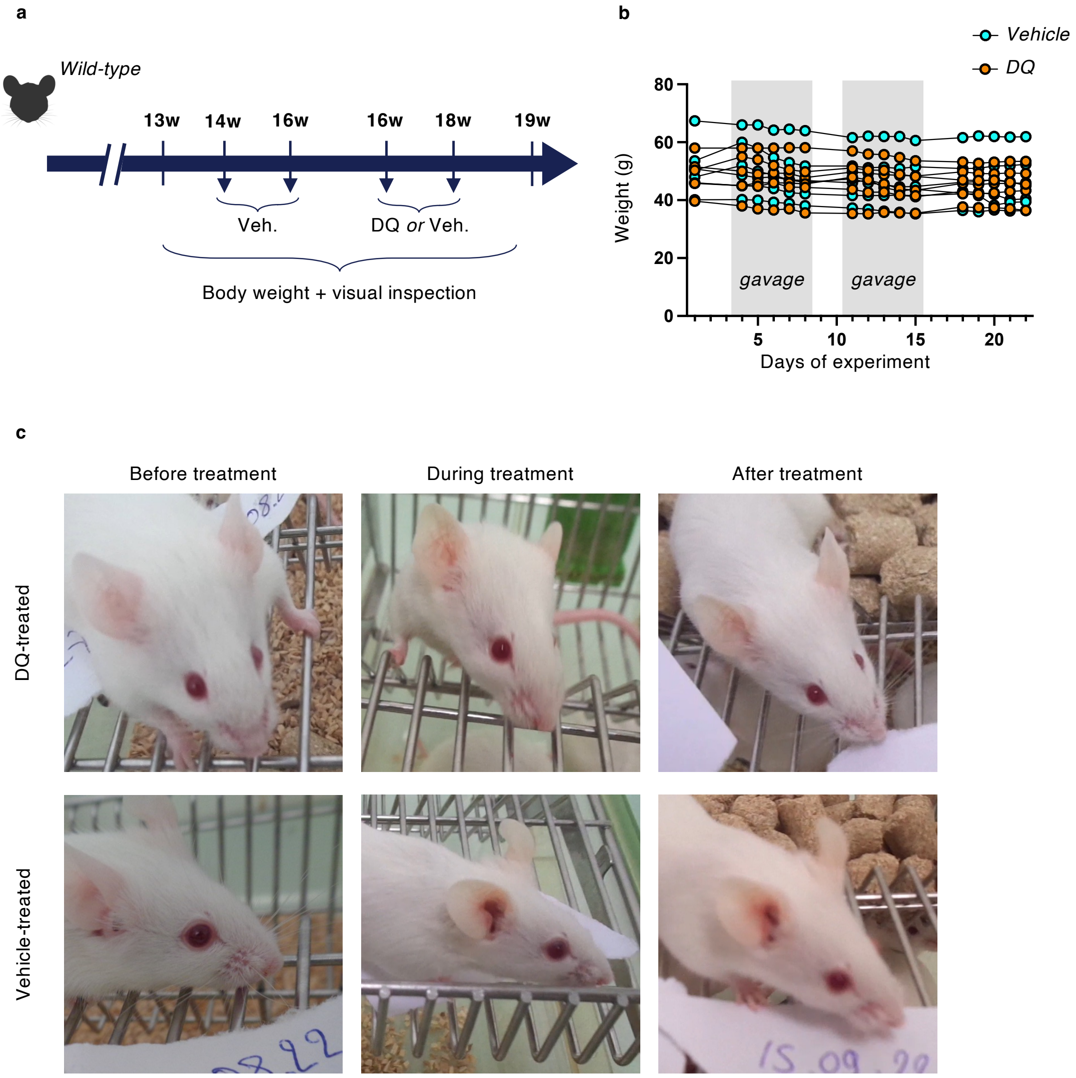

Supplementary Fig. 2. Absence of major side-effects in DQ-treated wild-type animals. (a) Study design for evaluation of potential deleterious effects following DQ administration. **(b)** Evolution of mouse body weight following DQ or vehicle administration by gavage over the course of two weeks on n=6 animals receiving either DQ or vehicle. **(c)** Representative images of facial indicators to assess animal well-being according to the mouse grimace scale.

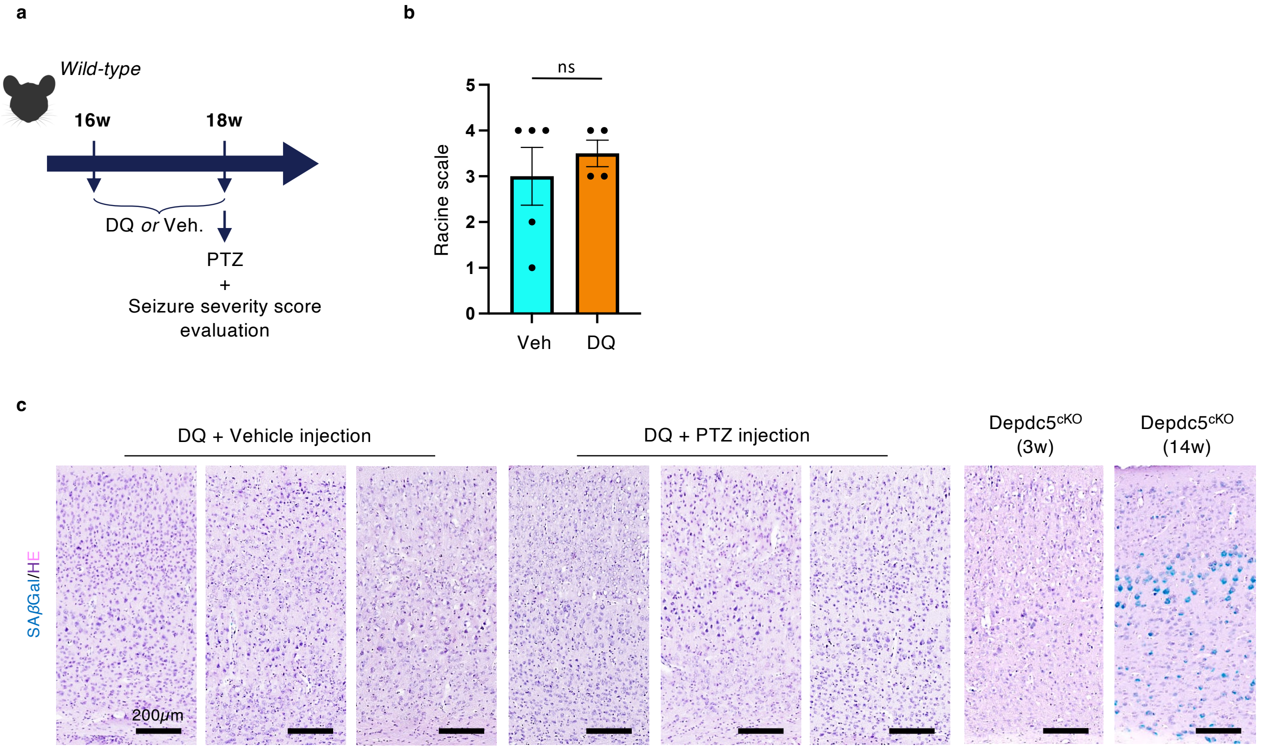

Supplementary Fig. 3. Absence of antiepileptic effects of DQ administration in a mouse model of pentylenetetrazol-induced seizures. (a) Study design for evaluation of potential antiepileptic effects following DQ administration. **(b)** Maximum seizure severity score reached based on Racine scale in n=5 animals who received vehicle and n=4 animals who received DQ (1: sudden behavioral arrest, 2: head nodding, 3: forelimb clonus with rearing, 4: generalized tonic-clonic seizure, 5: lethal seizure). Two-tailed Mann-Whitney test. ns: P=0.8413 **(c)** SAβGal colorimetric assay in pentylenetetrazol (PTZ)-induced seizures following vehicle or DQ administration. SAβGal colorimetric assay on a negative control (3 weeks-old Depdc5^{cKO}) and a positive control (14 weeks-old Depdc5^{cKO}). Scatter dot plots are presented as mean ± SEM. Colorimetric assay was repeated at least two times.

Supplementary Table 1. Detailed clinical features of the patient cohort

Patient ID	Neuropathology	Sex	Seizure frequency	Duration of epilepsy	Engel score	Anti-seizure medication	Used for experiments
**Cases**							
1 (FCD-64)	FCDIIb	M	Daily	2.5y	1	VPA, CBZ	IHC, SAβGal
2 (FCD-33)	FCDIIa	M	Weekly	5.6y	1	CBZ	IHC, SAβGal
3 (FCD-56)	FCDIIb	F	Daily+	15.4y	1	ZNS, TPM, OXC, LTG	IHC, SAβGal, LCM, EM
4 (FCD-57)	FCDIIb	M	Daily+	8.7y	3	LTG, OXC	EM
5 (FCD-59)	FCDIIb	M	Daily+	11.7y	2	CLB, OXC	IHC, SAβGal
6 (FCD-61)	FCDIIb	M	Weekly	6.5y	2	CBZ, STP, VGB	IHC, SAβGal
7 (FCD-70)	FCDIIb	M	Daily	13.6y	2	CLB, CBZ, TPM, FBM	EM
8	FCDIIb	M	Daily	N/A	1	LEV, VPA, CBZ, CLB	IHC, MEA
9	FCDIIb	M	Daily	28y	1	CBZ, LCS, CLB	MEA
10 (FCD-36)	HME/IIa	F	Daily+	2.4m	3	CBZ, LEV, VGB	IHC, SAβGal
11 (HME-73)	HME/IIa	F	Daily+	2.4m	1	CBZ, ZNS, LKZ, LEV	EM
12 (HME-74)	HME/IIa	M	Daily+	1.3y	1	LCS, VPA	SAβGal, EM
13 (HME-77)	HME/IIa	M	Daily	6m	1	TPM, VGB, CLB	IHC, SAβGal, LCM
14 (HME-79)	HME/IIb	M	Daily+	4.8m	1	VPA, TPM, CBZ, CLB	IHC, SAβGal
21	FCDIIa	M	Daily	7y	1	CBZ, LEV	IHC, SAβGal
22	FCDIIb	F	Daily	4y	2	LCS, OXC	IHC, SAβGal
23	FCDIIb	M	Weekly	6.6y	1	LCS, OXC, ZNS	IHC, SAβGal
24	FCDIIb	F	Daily	3.7y	1	CBZ, LCS	IHC, SAβGal
25	FCDIIb	M	Daily	6.3y	1	CBZ	IHC, SAβGal
26	FCDIIb	M	Daily	4y	3	TPM, RIS	IHC, SAβGal
27 (FCD-65)	FCDIIb	M	Daily	2.8y	1	LTG, VPA	IHC, SAβGal
28	FCDIIb	F	Daily	5y	1	LEV, VGB	IHC, SAβGal
29	FCDIIa	F	Daily	3.4y	1	OXC, TPM, VGB	IHC, SAβGal
30	FCDIIb	F	Daily	11y	1	CBZ, TPM	IHC, SAβGal
**Epileptic controls**							
15 (FCD-16)	FCDI	F	Daily+	2y	2	OXC, LEV	IHC, SAβGal
16 (FCD-13)	mMCD	M	Weekly	8.8y	3	VPA	IHC, SAβGal

17 (FCD-18)	FCDI	F	Daily	3.4y	1	OXC, LTG, CLB	IHC, SAβGal
18 (FCD-7)	mMCD	F	Daily+	5.3y	2	CLB, CBZ	IHC, SAβGal
19 (FCD-12)	FCDI	M	Weekly	3.5y	3	LCS, OXC	IHC, SAβGal
20 (FCD-6)	mMCD	F	Daily+	1.7y	3	VPA, LEV, VGB	IHC, SAβGal
31 (FCD-17)	FCDI	F	Daily	2m	1	CZP, VPA	IHC, SAβGal
32 (FCD-5)	mMCD	F	Daily	3y	1	CLB, LCS, OXC	IHC, SAβGal
33 (FCD-8)	mMCD	F	Daily	9.7y	2	CBZ	IHC, SAβGal
34 (FCD-15)	mMCD	M	Daily	6.3y	1	OXC, VPA	IHC, SAβGal
35	mMCD	F	Daily	3.1y	3	LEV, VGB	IHC, AβGal
36	mMCD	M	Daily	6y	1	CBZ, STP	IHC, SAβGal
37 (FCD-10)	FCDI	M	Daily	5.1y	1	LCS, OXC	IHC, SAβGal

Abbreviations: HME: hemimegalencephaly; mMCD: mild malformation of cortical development with excessive heterotopic neurons; m: months; y: years; duration of epilepsy: duration between seizure onset and surgery; Daily+: multiple per day. Seizure frequency is based on long-term EEG recordings. Anti-seizure medication abbreviations: CBZ: carbamazepine; CLB: clobazam; CZP: clonazepam; FBM: felbamate; LEV: levetiracetam; LCS: lacosamide; LKZ: licozam; LTG: lamotrigine; OXC: oxcarbazepine; RIS: risperidone; STP: stiripentol; VGB: vigabatrin; VPA: valproic acid; TPM: topiramate; ZNS: zonisamide. IDs in parentheses refer to patients previously reported in Baldassari et al [11]. Column 'Used for exp' indicates for which type of experiment samples were used; ICC: immunohistochemistry, SAβGal: SaβGal colorimetric assay; EM: electron microscopy; LCM: laser-capture microdissection followed by ddPCR; MEA: multielectrode array.

Supplementary Table 2. Descriptive statistics of the patient cohort

	**Age at surgery**	**Age at seizure onset**	**Duration of epilepsy**	**Seizure frequency**
**Cases**	8.45y (9.22y)	1.99y (3.22y)	6.45y (6.35y)	2.17 (0.64)
**Controls**	7.67y (4.78y)	3.22y (4.11y)	4.47y (2.75y)	2.08 (0.64)
**P value**	P=0.7364	P=0.3586	P=0.2042	P=0.6871

Descriptive statistics of four clinical parameters in the patient (n = 37) cohort. Data are presented as mean and SD between parentheses. For seizure frequency, a score of 1 was attributed for 'Weekly', a score of 2 for 'Daily' and a score of 3 for 'Daily+'. No statistical difference was observed between cases and controls by two-tailed unpaired t-test with Welch's correction.

Supplementary Table 3. List of differentially produced cytokines in *Depdc5*^{cKO} mice

Analytes	Canonical SASP	mTOR dependent	Diff. produced cKO vs. WT	
			5 weeks	10 weeks
**IL1b**	**X**	**X**	P=0.3939 ns	**P=0.0022 ****
**IL6**	**X**	**X**	P=0.2576 ns	**P=0.0303 ***
**CXCL1**	**X**	**X**	P=0.5887 ns	**P=0.0022 ****
**CXCL2**	**X**	**X**	P=0.1320 ns	**P=0.0043 ****
**CXCL10**	**X**	**X**	**P=0.0260 ***	**P=0.0022 ****
**CCL2**	**X**	**X**	P=0.6991 ns	**P=0.0022 ****
**CCL20**	**X**	**X**		**P=0.0195 ***
**CCL3**	**X**		P=0.3095 ns	**P=0.0022 ****
**IL16**			P>0.9999 ns	**P=0.0260 ***
**IL17A**			P=0.4848 ns	P=0.4156 ns
**IL17C**			P=0.2403 ns	**P=0.0368 ***
**IL21**			P=0.0568 ns	**P=0.0173 ***
**IL22**			P=0.3723 ns	**P=0.0152 ***
**IL33**				
**TNFa**				**P=0.0238 ***

The following analytes (n =14/29) were not detected in electrochemiluminescent assay: IL15, IFNg, IL2, IL4, IL5, IL9, IL10, IL12, IL17E, IL17F, IL23, IL27, IL30, IL31. Statistics: two-tailed Mann-Whitney test.

Supplementary Table 4. List of differentially produced cytokines in Mtor^{S2215F} mice

Analytes	Canonical SASP	mTOR dependent	Diff. produced mTOR^{S225F} vs. GFP
**IL1b**	**X**	**X**	**P=0.0022 ****
**IL6**	**X**	**X**	**P=0.0173 ***
**CXCL1**	**X**	**X**	**P=0.0022 ****
**CXCL2**	**X**	**X**	**P=0.0022 ****
**CXCL10**	**X**	**X**	**P= 0.0022 ****
**CCL2**	**X**	**X**	**P=0.0022 ****
**CCL3**	**X**		**P=0.0260 ***
**IL16**			**P=0.0022 ****
**IL17A**			P=0.9372 ns
**IL17C**			P=0.6688 ns
**IL33**			P=0.0649 ns
**TNFa**			**P=0.0022 ****

The following analytes (n = 17/29) were not detected in electrochemiluminescent assay: CCL20, IL15, IFNg, IL2, IL4, IL5, IL9, IL10, IL12, IL17E, IL17F, IL21, IL22, IL23, IL27, IL30, IL31. Statistics: two-tailed Mann-Whitney test.
