# Supplementary material for: Targeting pathological cells with senolytic drugs reduces seizures in neurodevelopmental mTOR-related epilepsy
Source: Nat Neurosci. 2024 May 6;27(6):1125–36. doi: 10.1038/s41593-024-01634-2 (PMC11156583; doi:10.1038/s41593-024-01634-2)
Supplement: Supplementary file 2 — Reporting Summary [file 41593_2024_1634_MOESM2_ESM.pdf]

nature portfolio

Corresponding author(s): Stephanie Baulac

Last updated by author(s): Mar 13, 2024

Reporting Summary

Nature Portfolio wishes to improve the reproducibility of the work that we publish. This form provides structure for consistency and transparency in reporting. For further information on Nature Portfolio policies, see our Editorial Policies and the Editorial Policy Checklist.

Statistics

For all statistical analyses, confirm that the following items are present in the figure legend, table legend, main text, or Methods section.

n/a	Confirmed	
☐	☒	The exact sample size (*n*) for each experimental group/condition, given as a discrete number and unit of measurement
☐	☒	A statement on whether measurements were taken from distinct samples or whether the same sample was measured repeatedly
☐	☒	The statistical test(s) used AND whether they are one- or two-sided<br>*Only common tests should be described solely by name; describe more complex techniques in the Methods section.*
☒	☐	A description of all covariates tested
☒	☐	A description of any assumptions or corrections, such as tests of normality and adjustment for multiple comparisons
☐	☒	A full description of the statistical parameters including central tendency (e.g. means) or other basic estimates (e.g. regression coefficient) AND variation (e.g. standard deviation) or associated estimates of uncertainty (e.g. confidence intervals)
☐	☒	For null hypothesis testing, the test statistic (e.g. *F*, *t*, *r*) with confidence intervals, effect sizes, degrees of freedom and *P* value noted<br>*Give P values as exact values whenever suitable.*
☒	☐	For Bayesian analysis, information on the choice of priors and Markov chain Monte Carlo settings
☒	☐	For hierarchical and complex designs, identification of the appropriate level for tests and full reporting of outcomes
☒	☐	Estimates of effect sizes (e.g. Cohen's *d*, Pearson's *r*), indicating how they were calculated

Our web collection on statistics for biologists contains articles on many of the points above.

Software and code

Policy information about availability of computer code

Data collection	In vitro electrophysiological data were acquired with Multi Channel Experimenter software (MultiChannelSystems Version 2.20).
Data analysis	In vitro electrophysiological data from MEA analysis were analyzed offline using custom algorithms written in Matlab (MathWorks) available on GitHub at this link: https://github.com/jcponcer/PoncerLab/tree/740189ac6b202c2182daad7730f4dbfb1d80118e/matlab_interictal_detection. SigmaPlot 13.1 (SPSS) was used for statistical analysis and graph generation.<br><br>Mouse EEG data were obtained/analyzed using Deltamed (Natus) and MATLAB (MathWorks; version R2015b).<br><br>Histological stainings were analyzed using: FiJi (ImageJ2, version 2.9.0/1.53t), NDP.view 2 (Hamamatsu).<br><br>Statistics were performed with GraphPad Prism10 for macOS (version 10.1.1 (270)).

For manuscripts utilizing custom algorithms or software that are central to the research but not yet described in published literature, software must be made available to editors and reviewers. We strongly encourage code deposition in a community repository (e.g. GitHub). See the Nature Portfolio guidelines for submitting code & software for further information.

Data

Policy information about availability of data

All manuscripts must include a data availability statement. This statement should provide the following information, where applicable:

- Accession codes, unique identifiers, or web links for publicly available datasets
- A description of any restrictions on data availability
- For clinical datasets or third party data, please ensure that the statement adheres to our policy

Source data files are supplied for every Data Figure and Extended Data Figure. The volume of data generated for MEA-recordings by Matlab algorithms (MathWorks) does not allow for easy online posting, but they will be provided upon request to JC Poncer. Paxinos and Watson mouse brain atlas was used to determine coordinates of electrode implantation (Paxinos, G.W., C. The Rat Brain in Stereotaxic Coordinates (2005))

Research involving human participants, their data, or biological material

Policy information about studies with human participants or human data. See also policy information about sex, gender (identity/presentation), and sexual orientation and race, ethnicity and racism.

Reporting on sex and gender	FCDII is considered a non-gender-specific disease, therefore both males and females biological samples were included in this study.
Reporting on race, ethnicity, or other socially relevant groupings	There were no social, ethnic or racial categorization done in this study.
Population characteristics	All population characteristics are detailed in the manuscript (Table 1 and Supplementary tables 1 and 2), with descriptive features and statistics of the patient cohort.
Recruitment	Patient recruitment was based on 37 operated children for drug-resistant epilepsy (aged from 3 months to 16 years) at the Rothschild Foundation Hospital (Paris, France) between 2016 and 2020. The cohort consisted of cases with FCDII or hemimegalencephaly (HME) (n=24), and epilepsy surgical cases used as controls with a neuropathological diagnosis of FCDI (n=5), mild malformation of cortical development (mMCD=8). Patients were selected to ensure a homogenous cohort in terms of age at seizure onset, age at surgery, duration of epilepsy, seizure frequency, and surgical outcome (Supplementary Tables 1 and 2). No compensation was included.
Ethics oversight	The study protocol received ethical approval by the committees of CPP Ile de France II (N° ID-RCB/EUDRACT-2015-A00671-48) and the INSERM Ethics Review Committee (#22-879) by the INSERM Institutional Review Board (IRB00003888, IORG0003254, FWA00005831).

Note that full information on the approval of the study protocol must also be provided in the manuscript.

Field-specific reporting

Please select the one below that is the best fit for your research. If you are not sure, read the appropriate sections before making your selection.

☒ Life sciences ☐ Behavioural & social sciences ☐ Ecological, evolutionary & environmental sciences

For a reference copy of the document with all sections, see nature.com/documents/nr-reporting-summary-flat.pdf

Life sciences study design

All studies must disclose on these points even when the disclosure is negative.

Sample size	1/ Human work:<br>No statistical methods were used to pre-determine sample sizes as sample size is limited by the availability of human brain material<br>- For MEA analysis, we conducted electrophysiological recordings in 4 slices from one genetically-solved FCDII patient (#8) and replicated the findings in 2 slices of a second genetically-solved FCDII patient (#9).<br>- For histological stainings, we selected a panel of 30 flash-frozen tissues representative of the genetic etiology of FCDII (N=18) and matched controls (N=13), and of sufficient quality/preservation to conduct enzymatic assay and immunohistochemistry. No samples were excluded. This represents a large cohort of samples compared to similar other key studies (Lamparello et al. 2007 doi:10.1093/brain/awm175; Honke et al. 2023, doi.org/10.1186/s40478-023-01675-x; Orlova et al. 2010, doi: 10.1097/NEN.0b013e3181eac1f5).<br><br>2/ Mouse work:<br>- Depdc5 model: For colorimetric and immunostainings, between 4 and 6 mice/genotype/condition were used to allow non-parametric statistical analyses of acquired data. No sample size calculation was performed<br>- Mtor model: Administration of DQ or vehicle was performed on 6 mice/group for each condition to allow statistical testing with nonparametric Mann-Whitney tests that does not require assumption of normality distribution and accomodates limited sample size. No statistical methods were used to pre-determine sample sizes . Samples size was based on similar published studies (Nguyen et al 2023, doi.org/10.7554/eLife.91010.1; Lim JS, et al. Am J Hum Genet. 2017, doi: 10.1016/j.ajhg.2017.01.030), taking into account the inter-

individual variability inherent to the IUE procedure and ethical considerations
- For SASP analysis in both Depdc5 and Mtor models, we used 3 biological replicates and 2 technical replicates.
- For Western blotting, we used at least 3 biological replicates and at least 2 technical replicates.

Data exclusions	No data was excluded from experiments.
Replication	Depending on the experiment, we included 3 to 6 biological replicates. As shown in the manuscript, all attempts at replication were successful. Moreover, some experiments were interrupted by the COVID19 pandemics and replicates were performed several months apart, strengthening the robustness of our findings.
Randomization	1/ Human work:<br>Patient samples were selected based on genetic etiology, neuropathology diagnosis and tissue quality.<br>Both male and female animals were used.<br><br>2/ Mouse work:<br>In vivo study design was longitudinal and therefore no assignation to experimental groups were made.
Blinding	1/ Human work:<br>- For electrophysiological recordings, the experimenter was blind to the genotype of the patient tissue (presence or absence of mTOR-activating somatic mutations) during data collection and analysis<br>- In all stainings, the experimenter was blind to the phenotype (FCDII versus epileptic controls) and genotype of the patient tissue (presence or absence of mTOR-activating somatic mutations) during data collection and analysis<br><br>2/ Mouse work<br>- For video-EEG analyses, the experimenter was blind to the treatment protocol (DQ versus vehicle) during data collection and analysis<br>- For stainings, the experimenter was blind to the mice genotypes (mutant or WT) or to the treatment (DQ versus vehicle) during data collection and analysis

Reporting for specific materials, systems and methods

We require information from authors about some types of materials, experimental systems and methods used in many studies. Here, indicate whether each material, system or method listed is relevant to your study. If you are not sure if a list item applies to your research, read the appropriate section before selecting a response.

Materials & experimental systems

n/a	Involved in the study	
☐	☒	Antibodies
☒	☐	Eukaryotic cell lines
☒	☐	Palaeontology and archaeology
☐	☒	Animals and other organisms
☐	☒	Clinical data
☒	☐	Dual use research of concern
☒	☐	Plants

Methods

n/a	Involved in the study	
☒	☐	ChIP-seq
☒	☐	Flow cytometry
☒	☐	MRI-based neuroimaging

Antibodies

Antibodies used

Primary antibodies for immunostainings:
p53 (1:300; DAKO #M7001, mouse, DO-7), p16 (1:200; Abcam #108349, rabbit, EPR1473), pS6 S240/244 (1:1000; CST #5364, rabbit,D68F8 lot 8), SMI311R (1:400; BioLegend #837801, mouse, lot B284375), VIM (1:400; DAKO #M0725, mouse, V9), p21 (1:200; Abcam #ab188224, rabbit, EPR18021), Hmgb1 (1:100; CST #6893; rabbit, D3E5, lot 2), LaminB1 (1:100; CST #17416; rabbit, E6M5T, lot 1), NeuN (1:500; Merck #MAB377, mouse, A60, lot 2639366, 3104227 and 3832727), Olig2 (1:200; Abcam #ab109186, rabbit, EPR2673, lot GR210294-21| 1:100; Merck #MABN50, mouse, 211F1.1 lot 3859535 and 1028563.1), GFAP (1:300; LifeTechnologies #MA5-15086; mouse,54D2, lot 4 and 13).

Secondary antibodies for immunostainings:
Alexa 555 anti-rabbit (1:1000, Thermo fischer # A31522, lot 2339822), Alexa 488 anti-mouse (1:1000, Thermo fischer #A21202, lot 2147618), Biotinyled anti mouse (1:250, Vector laboratory # BA-2000, lot ZH0412), Biotinyled anti rabbit (1:250, Vector laboratory # BA-1100, lot ZH0421)

Primary antibodies for Western blotting:
Depdc5 (1:250; Abcam #ab185565; rabbit, EPR20497-23, lot GR3249313-5 and 1028563-1), p53 (1:300; DAKO #M7001, mouse, DO-7), p19 (1:2; CNIO; rat, PIL346C), Actin (1:1000; Merck #A2066; rabbit, lot 103M4826V and 095M4765V), pS6 S240/244 (1:1000; CST #5364, rabbit,D68F8 lot 8), total ribosomal protein S6 (1:1000; CST #2317S; mouse, 54D2, lot 4 and 13)

Secondary antibodies for Western Blotting:
anti-rabbit-HRP: Cell Signaling 7074 at 1/2000; anti-mouse-HRP: Cell Signaling 7076 at 1/2000; anti-rat-HRP: Cell Signaling 7077 at 1/2000.

Validation	According to the manufacturers website :<br>The mouse p53 antibody (M7001) has been cited in 614 publications<br>The rabbit p16 antibody (108349) has been cited in 223 publications<br>The rabbit pS6 S240/244 antibody (5364) has been cited in 808 publications<br>The mouse SMI311R antibody (837801) has been cited in 18 publications<br>The mouse Vimentin antibody (M0725) has been cited in 600 publications<br>The rabbit p21 antibody (ab188224) has been cited in 183 publications<br>The rabbit Hmgb1 antibody (ab188224) has been cited in 121 publications<br>The rabbit LaminB1 antibody (ab188224) has been cited in 19 publications<br>The mouse NeuN antibody (ab188224) has been cited in 6 536 publications<br>The rabbit Olig2 antibody (ab109186) has been cited in 193 publications<br>The mouse Olig2 antibody (MABN50) has been cited in 294 publications<br>The mouse GFAP antibody (MA5-15086) has been cited in 11 publications<br>The rabbit Depdc5 antibody (ab185565) has been cited in 7 publications<br>The rabbit actin antibody (A2066) has been cited in 334 publications<br>The mouse total ribosomal protein S6 antibody (2317S) has been cited in 814 publications<br><br>Anti-p19 antibody reactivity validation was provided by CNIO (unpublished but available through CNIO).<br>Specificity of the Depdc5 antibody was validated using Depdc5 ko lysates (Ann Neurol; 2022; doi: 10.1002/ana.26256)

Animals and other research organisms

Policy information about studies involving animals; ARRIVE guidelines recommended for reporting animal research, and Sex and Gender in Research

Laboratory animals	Strains used are:<br>1/ Depdc5flox/flox;Syn-Cre+/- (Depdc5 cKO) and Depdc5flox/flox;Syn-Cre-/- (Depdc5 WT) on a C57BL6/J background. They were obtained from breeding between Depdc5flox/flox mice and Synapsin1-Cre mice (B6.Cg-Tg(Syn1-cre)671Jxm/J, JAX N° 003966) mice .<br>Depdc5 cKO mice are previously described in Bacq et al. (Ann Neurol; 2022; doi: 10.1002/ana.26256).<br><br>2/ MtorS2215F mice were generated by in utero electroporation (IUE) at E14.5 in wild-type Swiss/CD1 (janvier Labs)
Wild animals	No wild animals were used in the study.
Reporting on sex	Both female and male animals were used in this study. Epileptogenic FCD affects both men and women without distinction, we therefore used animals from both sex to model the disease. In utero electroporation efficiency and phenotype penetrance is the same, regardless of the sex of the animal. We therefore dismissed any sex-bias in our study.
Field-collected samples	No field collected samples were used in the study.
Ethics oversight	The study protocol received ethical approval by the French Ministry of Research (no. APAFIS#26557, 37296, 40207). All mice were kept and bred under controlled conditions with 12/12 h light/dark cycle, 45-65% humidity, a temperature of 22°C as well as food and water ad libitum. All efforts were made to minimize the suffering and number of animals used in this study.

Note that full information on the approval of the study protocol must also be provided in the manuscript.

Clinical data

Policy information about clinical studies

All manuscripts should comply with the ICMJE guidelines for publication of clinical research and a completed CONSORT checklist must be included with all submissions.

Clinical trial registration	NCT02890641
Study protocol	Available on clinicaltrials.gov
Data collection	Patient recruitment was performed between 2016 and 2020 at the Fondation Ophtalmologique Adolphe de Rothschild and consisted in the collection of surgical brain specimen and blood samples from patients who underwent focal drug-resistant epilepsy surgery.<br>Data and analysis were performed between 2018 and 2023.
Outcomes	Not applicable, this clinical trial aims to collect samples.
